# Effects of leaf colorness, pigment contents and allelochemicals on the orientation of the Asian citrus psyllid among four Rutaceae host plants

**DOI:** 10.1186/s12870-019-1818-7

**Published:** 2019-06-13

**Authors:** Zao-Fa Zhong, Xiao-Juan Zhou, Jin-Bei Lin, Xin-Jun Liu, Jia Shao, Ba-Lian Zhong, Ting Peng

**Affiliations:** National Navel Orange Engineering Research Center, College of Life Sciences, Gannan Normal University, Ganzhou, China

**Keywords:** Huanglongbing, *Diaphorina citri*, wild citrus germplasm, behavioral analysis, attractants, repellents

## Abstract

**Background:**

Asian citrus psyllid (ACP) is the primary vector responsible for the transmission of the phloem-limited bacteria *Candidatus* Liberibacter spp., associated with huanglongbing (HLB), which causes great loss to the citrus industry. Although the roles of leaf color and volatile compounds in the orientation of ACP have been proven, the quantification of color and allelochemicals in the host plant are kept unclear, especially in wild citrus germplasms.

**Results:**

Chongyi wild mandarin significantly attracted more ACP than wild Hong Kong kumquat, ‘Gannan zao’ navel orange and orange jasmine did in the four-choice and olfactometer assays. The color parameters of the tender leaves from Chongyi wild mandarin and ‘Gannan zao’ were similar. The yellow color in both of them was less saturated than that of the other two plants species, but Chongyi wild mandarin had significant lower carotenoid content (*P* < 0.05). Notably metabolic profiling differences were observed among the healthy tender shoots from the four tested plants via UPLC-QQQ-MS and GC-MS analyses. Comparing with the other three plant species, 66 and 50 metabolites with significantly different contents in Chongyi wild mandarin were selected as UPLC-identified and GC-identified metabolites of interest (*P* < 0.05), respectively. Flavonoids accounted for a large group of secondary metabolites of interest, which may function as stimulants or repellents of ACP. Higher content of salicylic acid o-hexoside and lower content of (+)-jasmonic acid in Chongyi wild mandarin may lead to higher amount of methyl salicylate (an ACP attractant) and lower amount of trans-ocimene (an attractant to herbivores’ natural enemies) as well as the suppression of JA-mediated wounding response. This kind of synergistic or antagonistic effect among the metabolites differentially accumulated in Chongyi wild mandarin made it a more attractive host plant to ACP.

**Conclusions:**

Less saturated yellow color, high amount of attractants, low amount of repellents and insensitivity of JA-mediated wounding response are the four possible reasons why Chongyi wild mandarin attracted more ACP. This work may shed light on the olfactory and visual response of ACP to wild citrus germplasm hosts, and suggest the feasibility of developing ACP attractants or repellents patterned on potential metabolites.

**Electronic supplementary material:**

The online version of this article (10.1186/s12870-019-1818-7) contains supplementary material, which is available to authorized users.

## Background

Huanglongbing (HLB), also called citrus greening, is one of the most economically devastating diseases of citrus worldwide, which is caused by the phloem-limited fastidious bacteria “*Candidatus* Liberibacter asiaticus” (*C*Las), “*Ca.* L. africanus” and “*Ca.* L. americanus” [1]. Among these three species, *C*Las is believed to be the most dominant one and has caused huge economic losses to citrus industry globally [2]. At the early stage, infected trees show up blotchy-mottled or completely yellow chlorotic leaves and deformed small fruits with aborted seeds [3]. As the pathogen manipulates, the infected trees will suffer from the dysfunction of source-sink carbohydrate translocation, which leads to significant fruit-dropping and yield reduction [4]. Now there are no commercial citrus cultivars that are strongly tolerant to *C*Las [2, 5]. Numerous measures have been tested to address this dysfunction or control the manipulation of *C*Las, such as the application of enhanced foliar nutrition and thermotherapy [3, 6]. However, citrus growers mainly rely on three steps to reduce the incidence of HLB, namely eradication of infected trees, the use of healthy nursery stocks and control of the insect vector via pesticides [2, 7].

Although *C*Las can be transmitted via graft inoculation, Asian citrus psyllid (ACP), *Diaphorina citri* Kuwayama (Hemiptera: Liviidae), is believed to be the primary vector of the bacterial causative agent of HLB [2, 8]. The plant hosts of *D. citri* include *Murraya paniculata* (L.) Jack, *M. exotica* L., *M. koenigii* L. and nearly all the citrus species [9–11]. However, wild citrus species were rarely reported as the host of ACP or used in HLB study.

The overuse of pesticides application could bring ACP under control, but it could also cause severe environmental damage, insect resistance and food safety problem as well as adverse effect on ACP’ natural enemies [12–14]. Many environmental friendly alternatives have been suggested, such as essential oils, parasitic wasp, entomopathogenic fungi and additional tactics that affect the visual cues of ACP, such as reflective mulching and kaolin [15–21]. Given the fact that ACP could accurately detect, evaluate, and discriminate visual cues, such as hue, saturation level and outline of the object, yellow sticky cards have been proved to be an effective green approach to monitor and trap ACP [22–24]. Although it could also be conditioned to recognize a blue-colored probing substrate [22], *D. citri* is innately attracted to bright yellow and green colors in the human visible spectrum that might resemble the flushing shoots [25–27]. Moreover, the addition of ultra-violet light could enhance the attraction of *D. citri* to green or yellow colors [27, 28]. Even though the role of color in the orientation of the ACP has been substantially studied, the quantification of color and related allelochemicals in leaves are kept unclear.

Besides color stimuli, olfactory cues also are critical in the initiation of host plant selection process by *D. citri* [25]. Therefore, repellents or attractants patterned on volatile compounds have also been suggested to be a potential way in the protection of citrus plants against HLB, which has been introduced in the push-pull strategy for ACP management [29–31]. (E)-*β*-caryophyllene identified from guava [30], trisulfides (dimethyl trisulfide) from garlic chive leaves [32], terpenoids from the non-host cashew all had repellent effect on ACP [33]. These repellent volatiles were mainly identified from non-Rutaceae plants. As to attractant, analysis on curry leaf preferred by female ACP revealed 10 monoterpenes and four sesquiterpenes as well as two unknown compounds that may function as attractants [34]. The identified metabolites were quite few. To have a better implementation of the push-pull strategy, a large number of volatiles that can influence the behavior of ACP should be identified.

In this study, first we intended to identify which host plants was the most preferred one by ACP. And then combined metabonomics techniques were applied to study the metabolite differences among the tender shoots of the four tested plants. Our goal was to identify the potential volatile chemicals or metabolites responsible for attracting or repelling ACP, which could be useful in ACP detection and monitoring.

## Results

### Chongyi wild mandarin was the most preferred host of *D. citri* among the four tested Rutaceae plants in both four-choice and olfactometer tests

**Fig. 1:**
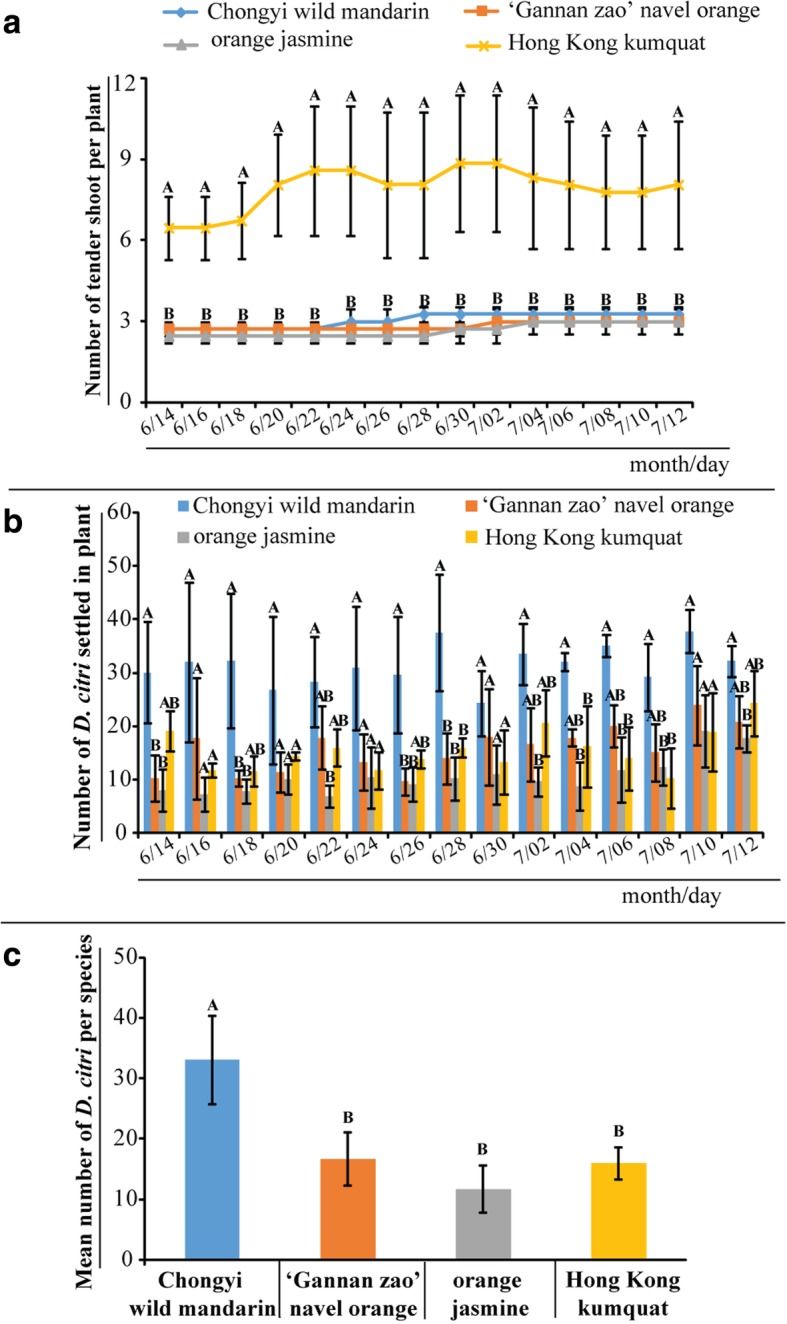
The numbers of tender shoot and *D. citri* attracted by each plant species in the four-choice test. I: the numbers of tender shoot in each plant species used in the four-choice test, which were counted at two days’ interval. II: the numbers of *D. citri* attracted by each host plant species in the four-choice test, which was repeated every two days and continued for one month; III: the total average number of attracted *D. citri* on each plant species in one month. Different letters marked above the bars indicated significant difference among the tested plants (*P* < 0.05). Except Chongyi wild mandarin, there was no significant difference among the tender shoot numbers from the other three plant species and the lines overlapped, so only one letter “B” was marked

During the one-month observing period of the four-choice test, wild Hong Kong kumquat (*Fortune hindisii* Swingle, Y) had significantly more tender shoots, 7 – 10 per plant, than that of Chongyi wild mandarin (C), ‘Gannan zao’ navel orange (*Citrus sinensis*, G) and orange jasmine (*Murraya exotica* L., J), 2 – 3 per plant (Fig. 1a, *P* < 0.05). There were no significant differences in the mean numbers of tender shoots among G, J and Y at the 15 time points (Fig. 1a, *P* > 0.05). In addition, *D. citri* exclusively fed on the flushing shoots and tender leaves of G, J, and Y. But, besides the tender shoots of Chongyi wild mandarin, few *D. citri* also fed on the mature leaves of the upper half of the plant. The results of the four-choice test revealed that C always attracted more *D. citri* than the other three tested plants in every counting time point (Fig. 1b). Out of the 15 time points, there were four times that C attracted significantly more *D.citri* than G and Y did, but 10 times that significantly more than J did (Fig. 1b, *P* < 0.05). Totally, the mean number of *D. citri* attracted by C in one month was significantly higher than the other three plant species at the significance level of *P* < 0.05 (Fig. 1c).

**Fig. 2:**
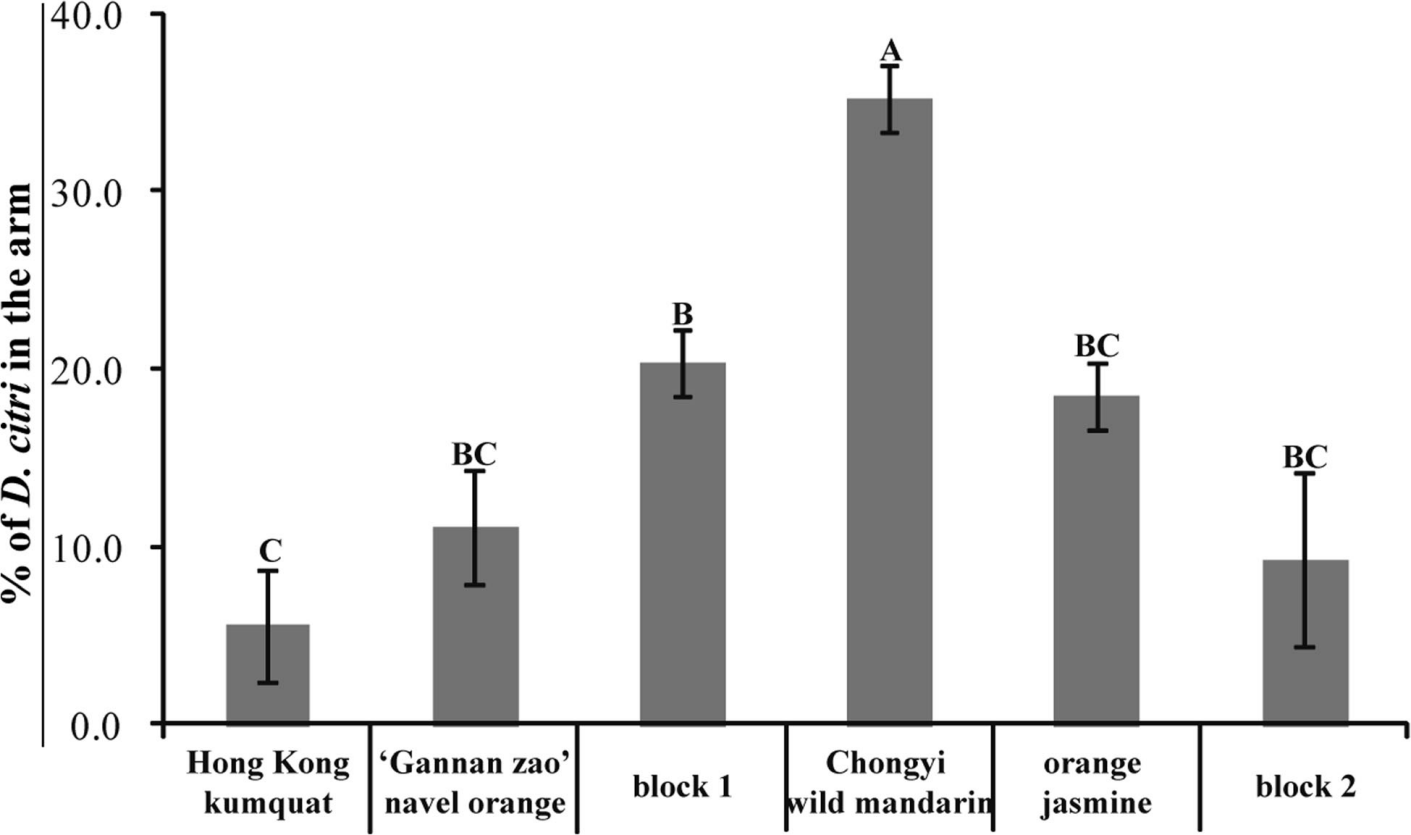
Percentage of female *D. citri* adults moved towards two blocked arms and four arms that were connected to the air source of Chongyi wild mandarin, ‘Gannan zao’ navel orange, orange jasmine, wild Hong Kong kumquat respectively, in the 6-arm olfactometer. Different letters marked above the bars indicated significant difference among the arms (*P* < 0.01)

In the 6-arm olfactometer with 2 arms blocked, the arm connected to the tender shoot of Chongyi wild mandarin always attracted significantly more female *D. citri* adults than the other five arms did (Fig. 2, *P* < 0.01). Due to peer effect, the two arms on the right and left sides of Chongyi wild mandarin always attracted more insects than the rest three arms. Similar results were obtained when orange jasmine, ‘Gannan zao’, wild Hong Kong kumquat was placed next to Chongyi wild mandarin one by one. Therefore, only one representative figure was showed. Since it was the most preferred host of *D. citri* among the four tested plant species, Chongyi wild mandarin was used as control in the subsequent experiments.

### Chongyi wild mandarin and ‘Gannan zao’ shared similar leaf color parameters

**Fig. 3:**
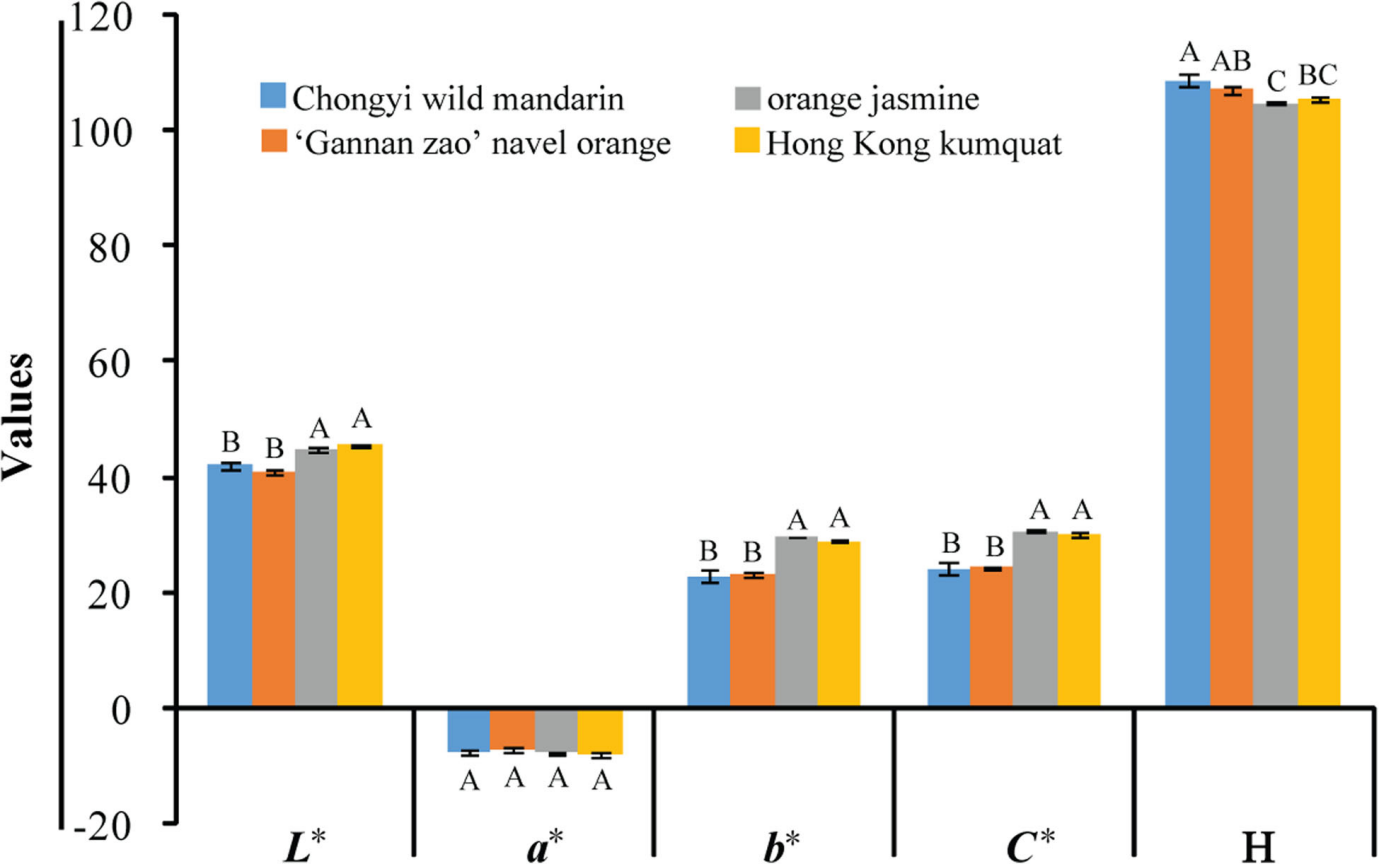
The differences of chromatic parameters among Chongyi wild mandarin, ‘Gannan zao’ navel orange, orange jasmine, wild Hong Kong kumquat. L^*^ = Lightness of the color, −a^*^ = Greenness, +b^*^ = Yellowness, C^*^ = Color saturation, *H*^∗^ = Color shading (60°= yellow; 120°= green). Different letters marked above the bars indicated significant difference among the tested plants (*P* < 0.05)

Color parameters of *L*^∗^, *a*^∗^, *b*^∗^, *C*^∗^ and *H*^∗^ from tender leaves were analyzed in order to check the leaf color differences among the four kinds of plants. The values of *a*^∗^ and *b*^∗^ in tender leaves of the four plants were negative and positive, respectively, suggesting that these leaves were green and yellow hue system (Fig. 3). In addition, the values of *H*^∗^ of these leaves ranged between 60° (yellow) and 120° (green), around 105° (Fig. 3), further proving that the leaves of all tested plants were the combination of green and yellow color. There was no significant difference among the values of *a*^∗^ (*P* > 0.05), which meant that all the tested leaves were fairly green (Fig. 3). The values of *L*^∗^, *b*^∗^, *C*^∗^ of tender leaves from C and G were significantly lower than that of J and Y (*P* < 0.05), suggesting that the tender leaves from J and Y were brighter, more yellowish and saturated than that of C and G (Fig. 3). The value of *H*^∗^ in tender leaves of C was highest and significantly higher than that of J and Y (Fig. 3, *P* < 0.05).

### The tender leaves of Chongyi wild mandarin had the lowest carotenoid content

**Fig. 4:**
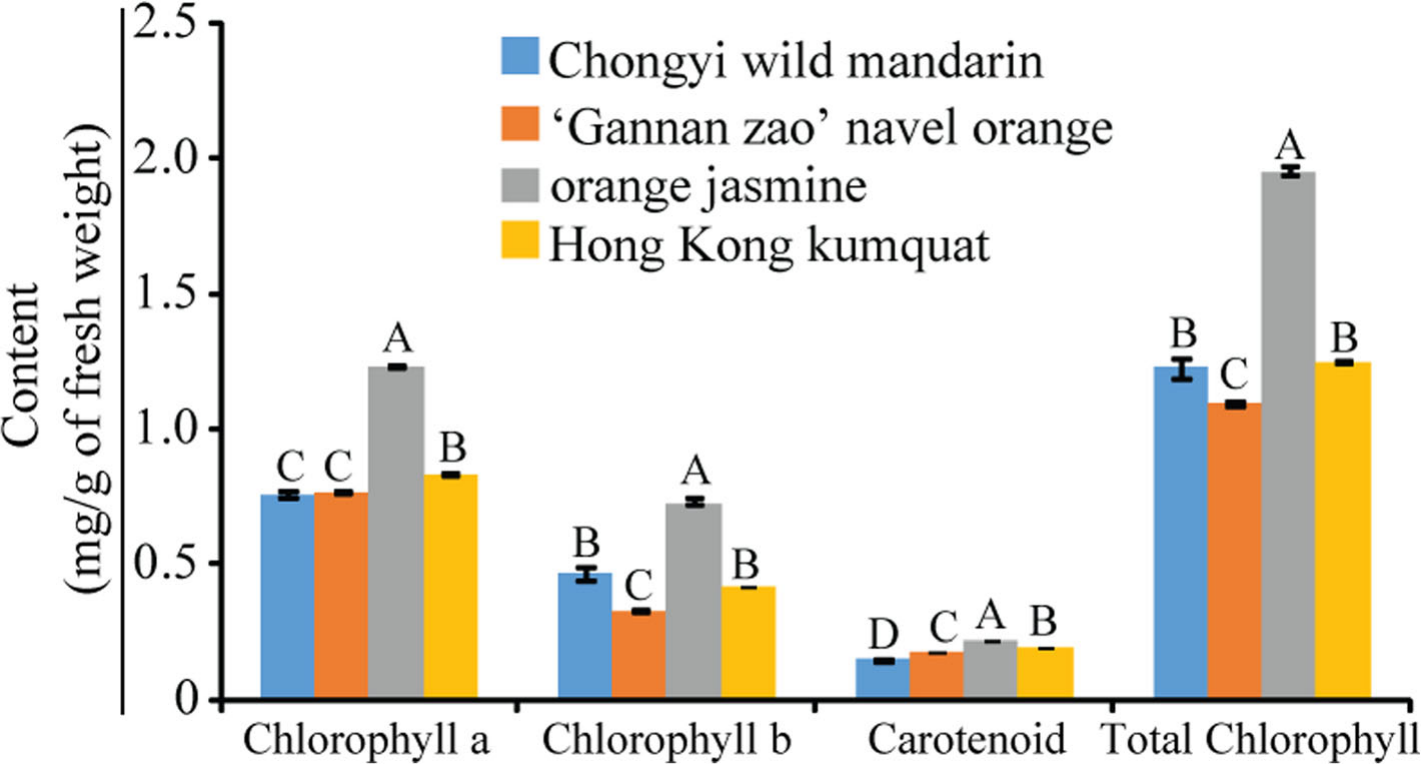
Chlorophyll and carotenoid contents in the tender leaves of Chongyi wild mandarin, ‘Gannan zao’ navel orange, orange jasmine, wild Hong Kong kumquat. Different letters marked above the bars indicated significant difference among the tested plants (*P* < 0.05)

The contents of Chlorophyll (Chl) *a*, Chl *b*, Carotenoid (Car) and total Chl in orange jasmine were significantly higher than that in Chongyi wild mandarin, ‘Gannan zao’ and wild Hong Kong kumquat at the significance level of *P* < 0.05, and Chongyi wild mandarin had the lowest Car content (Fig. 4).

### Untargeted metabolic profiling revealed allelochemicals with different contents between Chongyi wild mandarin and the other three plant species

The following results of ultra high performance liquid chromatography coupled to triple quadrupole tandem mass spectrometry (UPLC-QQQ-MS) and solid-phase microextraction (SPME) combined with gas chromatography-mass spectrometry (GC-MS) indicated that significant genotypic differences were observed in the metabolic profiles among the tender shoots of Chongyi wild mandarin (C), ‘Gannan zao’ navel orange (G) and orange jasmine (J) and wild Hong Kong kumquat (Y) under normal growth condition (Additional file 1: Figure S1 and Figure S2).

### UPLC-identified metabolic profiling

The non-biased UPLC-QQQ-MS detected totally 391 peaks, of which 126 were unknown metabolites and the remaining peaks were identified as known metabolites, such as eriodictyol, betaine, punicic acid and L-valine. Metabolites were highly reproducible among the three analyzed biological replications. Based on the principal component analysis (PCA) results, a separation of samples was observed and the first principal component (PC1) contributed 65.8%, 76.6%, 57.4% of variation in the data set of G/C, J/C, Y/C, respectively (Additional file 1 :Figure S1).

**Fig. 5:**
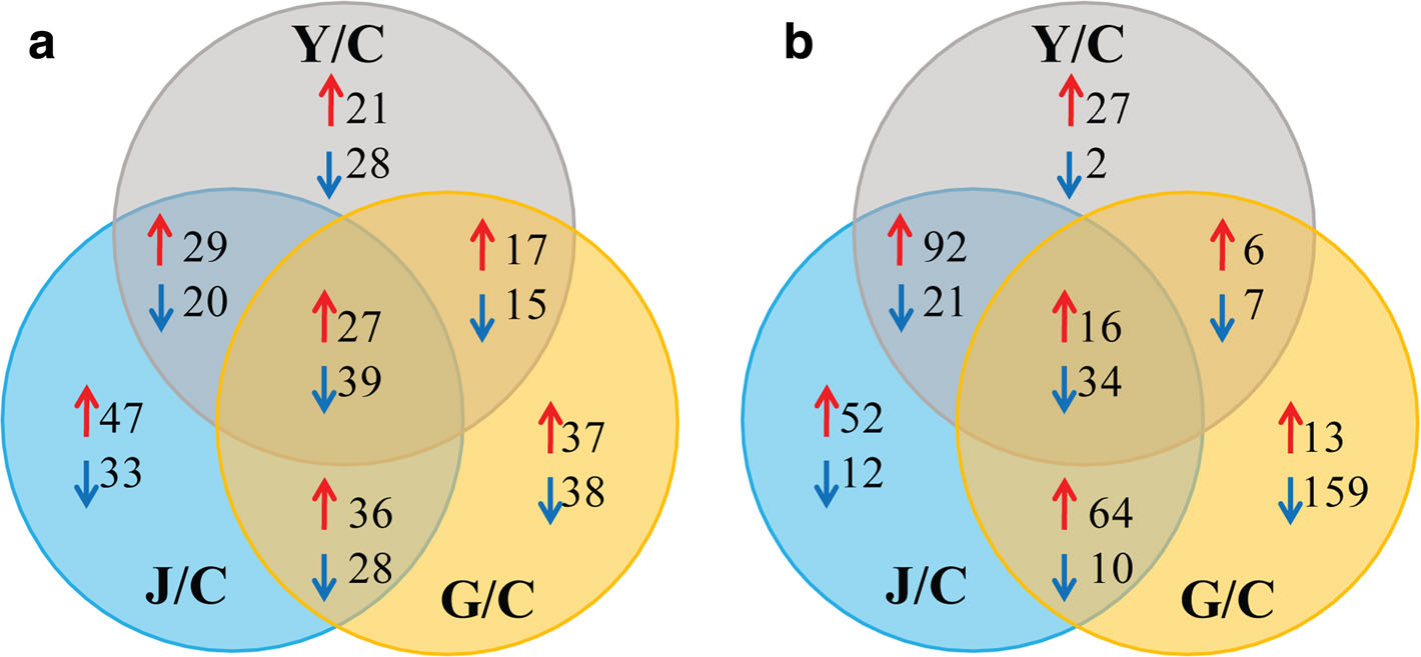
Venn diagram of the differentially accumulated metabolites from ‘Gannan zao’ navel orange (G), orange jasmine (J), wild Hong Kong kumquat (Y) comparing with Chongyi wild mandarin (C) in UPLC-QQQ-MS (I) and GC-MS (II) analysis. Red and blue arrows mean significantly higher and lower, respectively (VIP > 1 and *P* < 0.05). The numbers on the right of the arrows indicated the numbers of metabolites with significantly different content

**Fig. 6:**
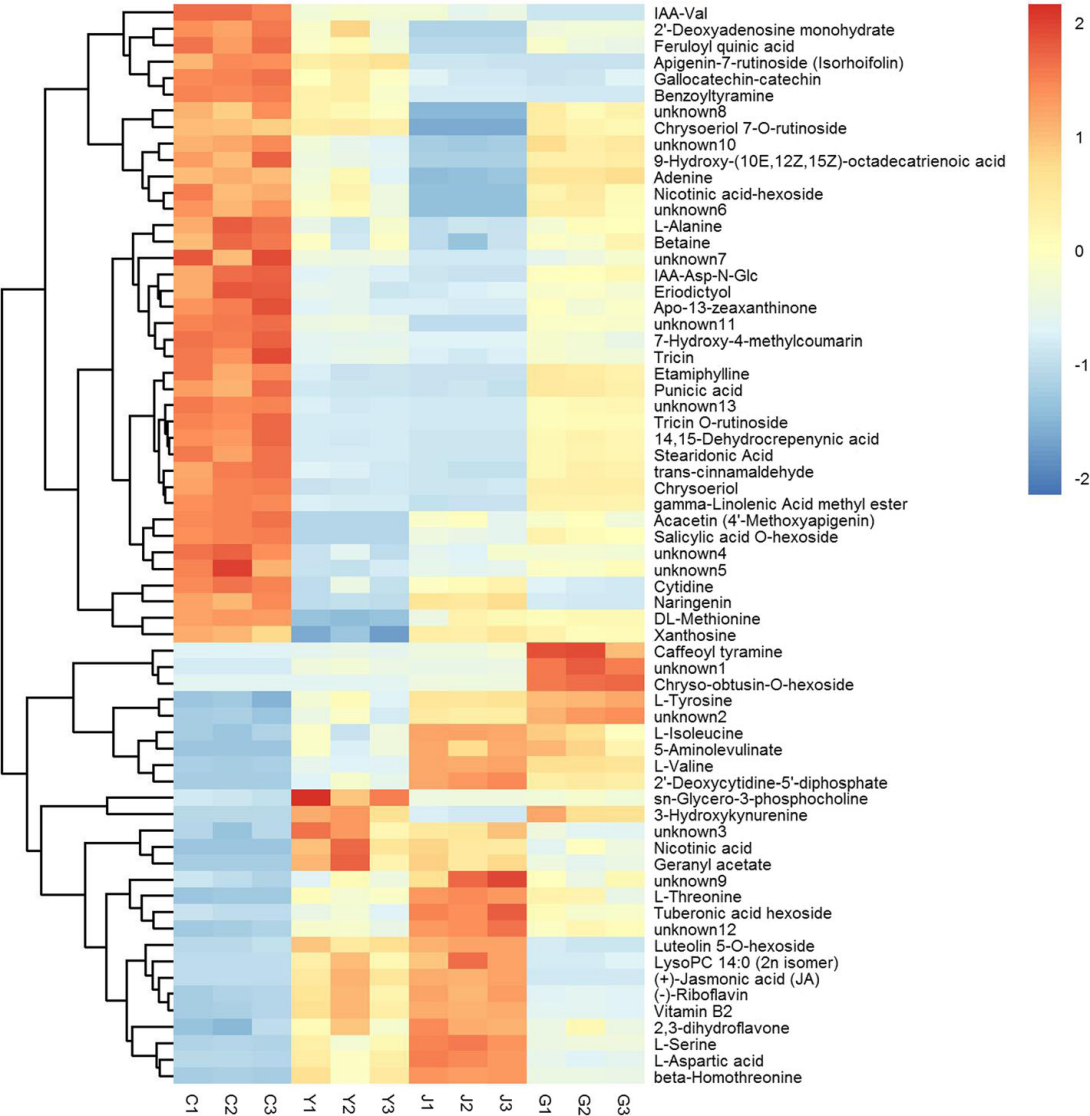
Heatmap illustrating levels of UPLC-identified metabolites of interest that were differentially accumulated in ‘Gannan zao’ navel orange (G), orange jasmine (J), wild Hong Kong kumquat (Y) comparing with Chongyi wild mandarin (C). The heatmap was generated via ‘Pearson’ and ‘Ward’ for distance measure and clustering algorithm, respectively. Columns represent biological replicates (n = 3 per group), and rows represent individual metabolites. The relative metabolite level is depicted according to the color scale. Red indicates higher level and green indicates lower level. The dendrograms indicate the overall similarity of metabolite expression profiles

To identify the important metabolites associated with host preference of ACP, the first principal component of variable importance in the projection (VIP) and Student's *t*-test were carried out. There were totally 237, 259, 196 secondary metabolites with significantly different (VIP > 1 and *P*-value < 0.05) content in G, J and Y, respectively, when C was used as the control (Table 1). As the Venn diagram shown in Fig. 5i, 66 UPLC-identified metabolites were differentially accumulated in G, J and Y when compared with C. The difference of their accumulation among the four plant samples was illustrated with heatmap, indicating that the content of each metabolite in every sample varied and the data had good repeatability among the replications (Fig. 6).

**Table 1:** Numbers of total detected metabolites and metabolites with significantly different contents (VIP > 1 and *P*-value < 0.05) in ‘Gannan zao’ navel orange (G), orange jasmine (J) and wild Hong Kong kumquat (Y) when Chongyi wild mandarin (C) was the control

Analysis	Combinations^1^	Numbers of total detected metabolites	Numbers of differentially accumulated metabolites		
			Up-accumulated	Down-accumulated	Total
UPLC-QQQ-MS	G /C	391	117	120	237
	J/C		139	120	259
	Y/C		94	102	196
HS-GC-MS	G /C	522	99	210	309
	J/C		224	77	301
	Y/C		141	64	205

^1^C was used as control in the data analysis

These 66 metabolites were listed in Table 2 as the UPLC-identified metabolites of interest. Amino acids were the largest differentially accumulated metabolite category, two with higher contents and seven with lower contents in C (Table 2), which explained that aminoacyl-tRNA biosynthesis was the most important pathway with significant enrichment (*P* < 0.05) in the pathway analysis (Additional file 1: Figure S3). To our limited knowledge, except L-tyrosine (an aromatic amino acid), trans-cinnamaldehyde, apo-13-zeaxanthinone and geranyl acetate, no other UPLC-identified metabolites of interest were directly aroma-related. Therefore, we focused on finding the color-related metabolites, such as chlorophyll, carotenoid, flavonoids.

**Table 2:** UPLC-identified metabolites with significantly different contents (VIP > 1 and *P*-value < 0.05) in ‘Gannan zao’ navel orange (G), orange jasmine (J) and wild Hong Kong kumquat (Y) when Chongyi wild mandarin (C) was used as control in data analysis

No.	Compounds	Class	VIP^1^			*P*-VALUE^2^			FOLD CHANGE^3^			LOG_FOLDCHANGE^4^		
			G/C	J/C	Y/C	G/C	J/C	Y/C	G/C	J/C	Y/C	G/C	J/C	Y/C
1	L-Alanine	Amino acids	1.176	1.131	1.153	1.64E-04	2.20E-04	1.62E-03	0.485	0.199	0.329	-1.044	-2.326	-1.605
2	DL-Methionine		1.216	1.005	1.209	3.76E-06	3.06E-02	2.11E-07	0.589	0.540	0.047	-0.764	-0.889	-4.417
3	Feruloyl quinic acid	Phenylpropanoids	1.181	1.137	1.184	2.86E-04	2.12E-03	7.47E-04	0.276	6.03E-05	0.372	-1.860	-14.02	-1.426
4	trans-Cinnamaldehyde		1.189	1.125	1.208	8.80E-04	5.18E-05	9.17E-05	0.519	0.040	0.117	-0.947	-4.635	-3.100
5	Gallocatechin-catechin	Catechin derivatives	1.185	1.125	1.195	1.07E-05	6.20E-06	4.22E-04	0.114	0.132	0.471	-3.139	-2.927	-1.087
6	Cytidine	Nucleotide and its derivates	1.207	1.109	1.155	2.37E-06	2.73E-04	2.55E-04	0.176	0.493	0.165	-2.507	-1.022	-2.598
7	Adenine		1.165	1.136	1.122	2.19E-03	1.92E-06	6.00E-03	0.898	0.423	0.676	-0.155	-1.241	-0.564
8	Xanthosine		1.153	1.038	1.209	3.83E-03	1.20E-02	1.36E-04	0.790	0.850	0.357	-0.340	-0.235	-1.488
9	2'-Deoxyadenosine monohydrate		1.205	1.135	1.047	8.58E-05	1.69E-03	2.41E-02	0.579	0.387	0.678	-0.789	-1.371	-0.560
10	Apigenin-7-rutinoside (Isorhoifolin)	Flavone	1.211	1.133	1.168	3.22E-03	3.24E-03	5.26E-03	6E-05	0.004	0.637	-14.12	-8.070	-0.651
11	Tricin O-rutinoside		1.206	1.137	1.226	3.15E-03	1.36E-03	1.30E-03	0.400	6E-04	0.014	-1.323	-10.60	-6.136
12	Chrysoeriol 7-O-rutinoside		1.161	1.137	1.189	1.96E-03	4.69E-04	1.40E-03	0.737	0.002	0.790	-0.441	-9.212	-0.340
13	Tricin		1.202	1.123	1.224	3.28E-04	1.16E-04	1.86E-04	0.264	0.051	0.145	-1.921	-4.284	-2.789
14	Chrysoeriol		1.197	1.136	1.228	8.07E-03	1.72E-03	2.21E-05	0.570	0.072	0.070	-0.811	-3.793	-3.845
15	Acacetin (4'-Methoxyapigenin)		1.187	1.057	1.231	1.06E-04	6.24E-04	4.20E-04	0.369	0.325	0.004	-1.439	-1.624	-8.055
16	Eriodictyol	Flavonoids	1.190	1.120	1.140	1.38E-03	4.59E-04	7.16E-04	0.318	0.094	0.123	-1.651	-3.413	-3.022
17	Naringenin		1.209	1.069	1.224	2.49E-03	6.51E-03	2.11E-03	0.092	0.722	0.018	-3.444	-0.471	-5.764
18	Benzoyltyramine	Tryptamines and its derivatives	1.211	1.136	1.135	1.48E-04	1.26E-04	1.32E-03	0.005	0.017	0.432	-7.694	-5.864	-1.211
19	Betaine	Alkaloids	1.153	1.101	1.105	4.03E-03	4. 09E-04	5.89E-03	0.586	0.257	0.490	-0.772	-1.958	-1.030
20	Etamiphylline		1.166	1.136	1.214	2.66E-03	3.31E-03	1.03E-04	0.618	0.115	0.144	-0.695	-3.126	-2.801
21	Apo-13-zeaxanthinone	Terpenoids	1.190	1.135	1.208	5.89E-04	4.50E-03	1.86E-04	0.319	0.061	0.109	-1.648	-4.038	-3.198
22	Nicotinic acid-hexoside	Vitamine related	1.139	1.137	1.135	6.11E-03	3.17E-03	5.19E-03	0.640	5E-04	0.485	-0.645	-11.09	-1.044
23	7-Hydroxy-4-methylcoumarin	Coumarins and its derivatives	1.169	1.137	1.225	5.86E-05	7.55E-04	4.93E-06	0.169	2E-04	0.049	-2.561	-12.15	-4.338
24	IAA-Asp-N-Glc	Indoles and its derivatives	1.182	1.133	1.213	1.77E-03	5.84E-03	3.76E-04	0.421	0.037	0.141	-1.25	-4.755	-2.831
25	14,15-Dehydrocrepenynic acid	Lipids-fatty acid	1.198	1.136	1.230	4.39E-04	2.35E-03	2.40E-03	0.481	0.039	0.069	-1.06	-4.67	-3.860
26	Stearidonic acid		1.198	1.136	1.229	4.52E-04	2.56E-03	2.63E-03	0.468	0.034	0.063	-1.094	-4.894	-3.993
27	γ-Linolenic acid methyl ester		1.209	1.136	1.229	1.35E-06	4.33E-08	9.69E-08	0.539	0.055	0.119	-0.893	-4.178	-3.072
28	Punicic acid		1.150	1.132	1.228	6.23E-03	4.59E-03	5.20E-03	0.638	0.110	0.138	-0.648	-3.178	-2.861
29	9-Hydroxy-(10E,12Z,15Z)-octadecatrienoic acid		1.142	1.135	1.211	9.60E-03	6.68E-03	1.09E-03	0.642	0.081	0.332	-0.640	-3.621	-1.593
30	IAA-Val	Phytohormones	1.211	1.109	1.225	7.21E-04	5.11E-05	1.82E-05	6E-04	0.183	0.249	-10.59	-2.450	-2.004
31	Salicylic acid O-hexoside		1.194	1.131	1.231	9.77E-05	5.95E-06	2.74E-04	0.479	0.232	2E-04	-1.062	-2.109	-12.50
32	unknown4	unknown	1.182	1.084	1.206	1.05E-04	4.25E-04	7.06E-05	0.129	0.262	0.349	-2.954	-1.932	-1.520
33	unknown5		1.173	1.125	1.217	4.14E-03	1.42E-02	7.93E-04	0.455	0.270	0.159	-1.136	-1.890	-2.649
34	unknown6		1.159	1.137	1.156	1.83E-03	1.55E-03	2.27E-03	0.472	4.5E-04	0.625	-1.083	-11.11	-0.679
35	unknown7		1.145	1.136	1.216	3.13E-03	1.40E-02	2.00E-02	0.184	3E-04	0.183	-2.44	-11.64	-2.449
36	unknown8		1.120	1.137	1.158	1.05E-02	3.84E-03	6.45E-03	0.680	1E-05	0.623	-0.556	-16.08	-0.683
37	unknown10		1.124	1.135	1.205	7.34E-03	1.81E-05	2.33E-04	0.738	0.086	0.352	-0.439	-3.547	-1.506
38	unknown11		1.209	1.137	1.227	3.65E-06	2.91E-04	3.48E-06	0.359	0.021	0.234	-1.478	-5.571	-2.098
39	unknown13		1.206	1.137	1.225	1.23E-05	2.91E-07	5.38E-07	0.455	0.060	0.073	-1.137	-4.054	-3.767
40	L-Isoleucine	Amino acids	1.125	1.181	1.109	7.56E-07	3.17E-03	3.36E-02	10.02	7.409	3.799	3.33	2.89	1.93
41	L-Aspartic acid		1.134	1.188	1.223	2.62E-06	1.72E-03	1.45E-02	26.67	5.677	14.31	4.74	2.51	3.84
42	beta-Homothreonine		1.135	1.206	1.225	3.90E-08	5.75E-07	1.77E-02	26.71	8.524	17.03	4.74	3.09	4.09
43	L-Valine		1.137	1.210	1.219	2.54E-07	3.59E-07	4.26E-04	5.154	4.233	1.969	2.37	2.08	0.98
44	L-Serine		1.136	1.207	1.224	4.77E-04	3.13E-05	1.74E-02	19.35	6.240	9.532	4.27	2.64	3.25
45	L-Threonine		1.136	1.193	1.228	3.65E-07	3.55E-02	5.05E-03	24.18	12.22	11.46	4.60	3.61	3.52
46	L-Tyrosine		1.130	1.205	1.147	1.52E-05	1.03E-05	1.19E-02	1.900	2.144	1.500	0.93	1.10	0.59
47	3-Hydroxykynurenine	Amino acid derivatives	1.136	1.211	1.231	2.27E-02	8.89E-03	9.43E-03	2.E+03	1.35E+04	2.E+04	10.75	13.72	13.88
48	Geranyl acetate	Phenylpropanoids	1.136	1.207	1.219	2.69E-03	4.60E-03	3.74E-02	13.79	5.873	15.88	3.79	2.55	3.99
49	sn-Glycero-3-phosphocholine	Cholines	1.113	1.184	1.219	1.62E-04	4.83E-04	1.88E-02	2.191	2.373	6.498	1.13	1.25	2.70
50	2'-Deoxycytidine-5'-diphosphate	Nucleotide and its derivates	1.137	1.211	1.229	8.43E-04	2.69E-04	3.62E-02	1.E+05	7.E+04	3.E+04	16.77	16.13	15.04
51	Luteolin 5-O-hexoside	Flavone	1.132	1.110	1.221	2.24E-06	8.97E-03	2.55E-04	10.55	1.753	8.409	3.40	0.81	3.07
52	2,3-dihydroflavone		1.103	1.096	1.138	1.12E-04	1.25E-02	1.11E-02	5.121	2.696	3.570	2.36	1.43	1.84
53	Chryso-obtusin-O-hexoside	Flavonoids	1.137	1.211	1.228	3.62E-04	2.71E-04	4.53E-02	5.E+03	4.58E+04	2.E+02	12.13	15.48	7.44
54	Caffeoyl tyramine	Tryptamines and its derivatives	1.135	1.210	1.228	3.78E-02	1.61E-02	3.86E-02	7.E+02	5.E+03	3.E+02	9.40	12.24	8.00
55	Nicotinic acid	Vitamine related	1.134	1.167	1.216	3.28E-03	3.26E-02	1.77E-02	2.810	1.915	3.294	1.49	0.94	1.72
56	(-)-Riboflavin		1.133	1.187	1.212	5.66E-06	1.93E-04	1.51E-02	5.091	1.879	4.114	2.35	0.91	2.04
57	Vitamin B2		1.134	1.192	1.217	4.14E-07	1.25E-04	8.57E-04	4.889	1.887	4.146	2.29	0.92	2.05
58	5-Aminolevulinate	Organic acid and its derivatives	1.135	1.204	1.203	3.84E-03	1.54E-02	4.24E-02	11.86	10.36	5.263	3.57	3.37	2.40
59	Tuberonic acid hexoside		1.125	1.182	1.171	2.87E-05	9.22E-04	1.13E-02	31.88	12.12	7.046	4.99	3.60	2.82
60	LysoPC 14:0 (2n isomer)	Lipids-glycerophospholipid	1.135	1.200	1.224	8.66E-03	1.37E-02	2.71E-02	37.29	4.857	24.98	5.22	2.28	4.64
61	(+)-Jasmonic acid (JA)	Phytohormones	1.137	1.206	1.224	2.00E-07	1.61E-05	1.13E-02	8.531	1.579	7.028	3.09	0.66	2.81
62	unknown1	unknown	1.137	1.211	1.230	1.58E-03	1.24E-03	1.25E-02	3.E+03	2.7E+04	5.E+03	11.69	14.74	12.21
63	unknown2		1.133	1.207	1.133	5.50E-06	1.50E-05	1.71E-02	2.099	2.677	1.531	1.07	1.42	0.61
64	unknown3		1.112	1.098	1.175	4.96E-04	8.97E-03	7.48E-03	2.500	1.523	2.813	1.32	0.61	1.49
65	unknown9		1.103	1.120	1.070	4.32E-03	1.02E-02	4.36E-02	6.443	3.003	2.524	2.69	1.59	1.34
66	Unknown12		1.134	1.199	1.205	9.71E-06	1.59E-04	8.56E-04	3.760	2.321	1.957	1.91	1.21	0.97

^1^VIP: the importance of the variable projection of the substance in the OPLS-DA model of the group.

^2^*P*-VALUE: the *P* value of the substance in the t test of the group.

^3^FOLD CHANGE: the multiplier relationship between the two groups of experiments in this group

^4^LOG_FOLDCHANGE: the logarithm of the Fold change to the base 2

The corresponding pathways were found in the KEGG analysis: porphyrin and chlorophyll metabolism, flavonoid biosynthesis pathway, flavone and flavonol biosynthesis pathway. In pathway analysis, the latter two pathways were singled out in the bubble plot (Additional file 1: Figure S3). Since KEGG analysis and pathway analysis used two different calculating methods, porphyrin and chlorophyll metabolism was not showed up in the bubble plot, in which 5-aminolevulinate (5-ALA), a pivotal compound in the biosynthesis of chlorophyll, had significantly lower content in C.

As the second largest UPLC-identified metabolite category with significant difference, eight flavones, six with higher content and two with lower content in C, were all yellow metabolites (Table 2). It was worth noting that three flavones with higher content in C (apigenin-7-rutinoside, tricin o-rutinoside and chrysoeriol 7-o-rutinoside) were all exclusive to Rutaceae. Eriodictyol and chryso-obtusin-o-hexoside were two yellow differentially accumulated flavonoids. Moreover, a yellow terpenoid (apo-13-zeaxanthinone) and a yellow phenylpropanoid (trans-cinnamaldehyde) were also identified. These differentially accumulated yellow compounds plus 5-ALA identified via UPLC-QQQ-MS analysis may play important roles in the attractiveness of C to ACPs.

### GC-identified metabolic profiling

525 peaks were detected and 522 GC-identified metabolites, namely volatile compounds (VOCs), could be left through interquartile range de-noising method in GC-MS analysis. The score scatter plot for PCA model showed that the original data of all samples were distributed within the 95% confidence interval of Hotelling’s *T*-squared ellipse (Additional file 1: Figure S2I). Chongyi wild mandarin, ‘Gannan zao’, orange jasmine and wild Hong Kong kumquat were separated by PC1, representing 68.2%, 71.7% and 77.5% of the variation, respectively (Additional file 1: Figure S2II, S2III and S2IV).

Comparing with C, there were totally 205, 309, 301 metabolites with significantly different (VIP > 1 and *P* < 0.05) content in G, J and Y, respectively (Table 1). G had 99 metabolites with significantly higher contents and 210 with significantly lower contents (Table 1). Near 74% and 68% metabolites’ contents in J and Y were significantly higher than that in C, respectively (Table 1).

Unlike the UPLC-identified metabolites, quite few GC-identified metabolites were involved in the KEGG analysis and pathway analysis. In the bubble plot of pathway analysis, pyruvate metabolism, sulfur metabolism and TAC cycle all showed up in the G/C, J/C and Y/C data set (Additional file 1: Figure S4), but actually they only had one or two hits. This result suggested that only one or two differentially GC-identified metabolites directly participated in these differentially metabolic pathways.

**Fig. 7:**
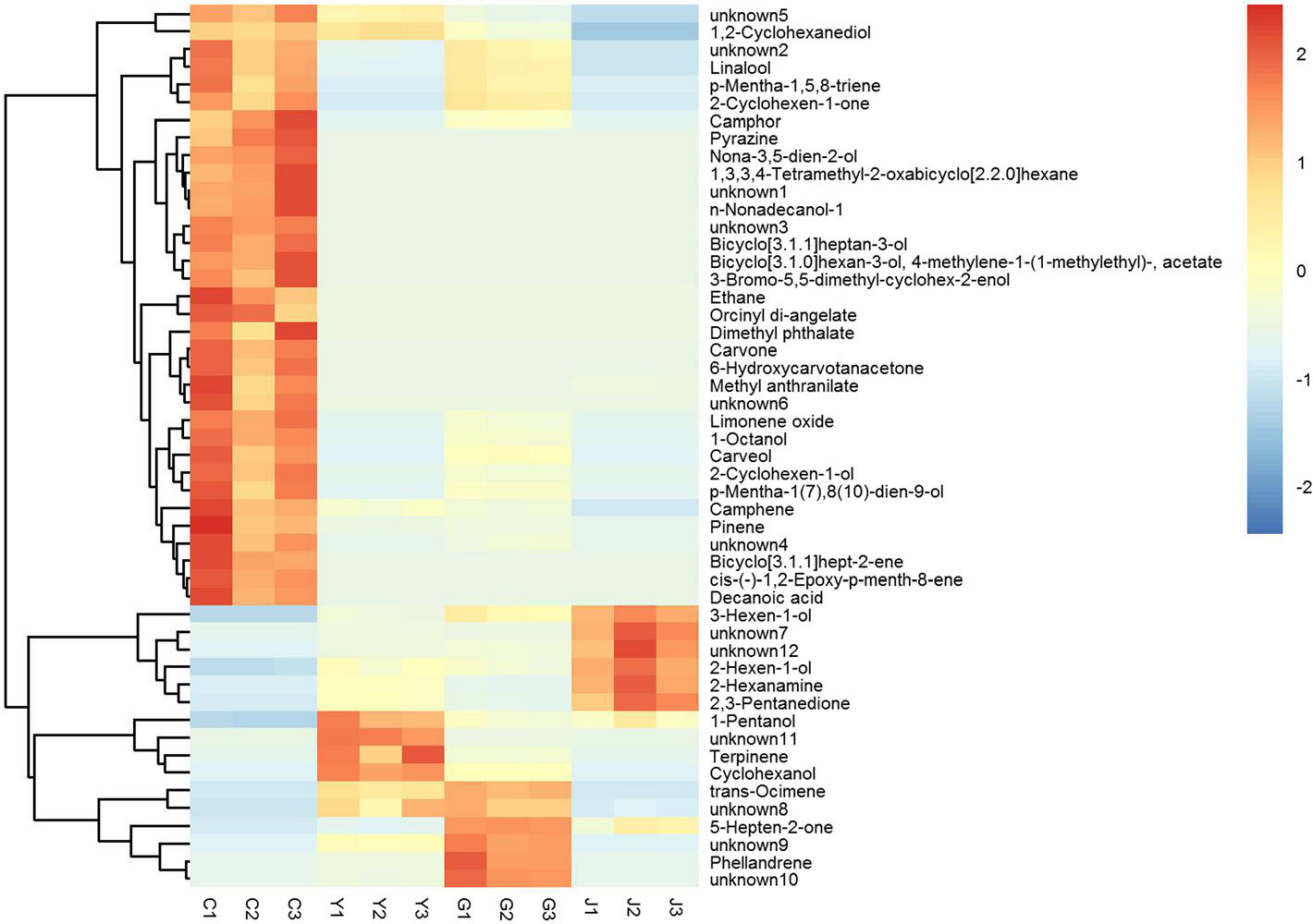
Heatmap illustrating levels of GC-identified metabolites of interest that were differentially accumulated in ‘Gannan zao’ navel orange (G), orange jasmine (J), wild Hong Kong kumquat (Y) comparing with Chongyi wild mandarin (C). The heatmap was generated via ‘Pearson’ and ‘Ward’ for distance measure and clustering algorithm, respectively. Columns represent biological replicates (n = 3 per group), and rows represent individual metabolites. The relative metabolite level is depicted according to the color scale. Red indicates higher level and green indicates lower level. The dendrograms indicate the overall similarity of metabolite expression profiles

As the Venn diagram showned in Fig. 5b, 50 VOCs were differentially identified, 34 with higher contents and 16 with lower contents in the tender shoot of Chongyi wild mandarin. The contents of these 50 VOCs in C, G, J and Y became evident in hierarchical clustering with heatmap (Fig. 7), which were selected as the GC-identified metabolites of interest and listed in Table 3. Intriguingly, except ethane and 12 unknown compounds, other 37 GC-identified metabolites of interest were all known aromatic compounds (Table 3). Monoterpenes accounted for the largest aromatic metabolite group, such as pinene, camphor, linalool, camphene, carveol, carvone and limonene, whose contents were significantly higher in Chongyi wild mandarin (Table 3). The levels of bicyclo [3.1.1] hept-2-ene, p-mentha-1,5,8-triene and cis-(-)-1,2-epoxy-p-menth-8-ene were also higher in Chongyi wild mandarin, comparing with the other three plants (Table 3). The odors released by those GC-identified metabolites with significantly higher contents in the VOCs of Chongyi wild mandarin may contribute to its attractiveness to ACP.

**Table 3:** GC-identified metabolites with significantly different contents (VIP > 1 and *P*-value < 0.05) in ‘Gannan zao’ navel orange (G), orange jasmine (J) and wild Hong Kong kumquat (Y) when Chongyi wild mandarin (C) was used as control in data analysis

No.	Compounds	ID^1^	Similarity^2^	VIP^3^			*P*-VALUE^4^			FOLD CHANGE^5^			LOG_FOLDCHANGE^6^		
				G/C	J/C	Y/C	G/C	J/C	Y/C	G/C	J/C	Y/C	G/C	J/C	Y/C
1	Bicyclo[3.1.1]hept-2-ene	30	696	1.191	1.167	1.341	0.015	0.015	0.015	1.38E-07	8.52E-07	2.91E-07	-22.8	-20.2	-21.7
2	Pinene	35	915	1.177	1.167	1.333	0.045	0.040	0.042	6.93E-02	4.35E-10	2.68E-02	-3.9	-31.1	-5.2
3	Camphene	49	928	1.167	1.167	1.305	0.031	0.018	0.033	2.42E-01	5.21E-08	2.93E-01	-2.0	-24.2	-1.8
4	Ethane	97	673	1.191	1.167	1.341	0.026	0.026	0.026	4.74E-08	2.92E-07	9.97E-08	-24.3	-21.7	-23.3
5	Unknown 1	104	834	1.112	1.166	1.341	0.014	0.014	0.014	1.46E-05	1.49E-08	5.08E-09	-16.1	-26.0	-27.6
6	Unknown 2	164	878	1.075	1.167	1.324	0.021	0.011	0.014	5.68E-01	1.49E-08	1.30E-01	-0.8	-26.0	-2.9
7	p-Mentha-1,5,8-triene	177	837	1.029	1.167	1.341	0.042	0.019	0.019	5.74E-01	5.25E-08	1.79E-08	-0.8	-24.2	-25.7
8	cis-(-)-1,2-Epoxy-p-menth-8-ene	188	790	1.191	1.167	1.341	0.011	0.011	0.011	8.13E-09	5.01E-08	1.71E-08	-26.9	-24.3	-25.8
9	Limonene oxide	193	941	1.183	1.167	1.341	0.005	0.005	0.005	1.56E-01	7.89E-09	2.69E-09	-2.7	-26.9	-28.5
10	Camphor	210	718	1.159	1.166	1.341	0.044	0.024	0.024	2.69E-01	6.56E-08	2.24E-08	-1.9	-23.9	-25.4
11	Pyrazine	215	726	1.191	1.166	1.341	0.020	0.020	0.020	1.10E-07	6.75E-07	2.30E-07	-23. 1	-20.5	-22.1
12	Linalool	227	975	1.097	1.167	1.335	0.017	0.010	0.012	5.89E-01	2.02E-10	1.09E-01	-0.8	-32.2	-3.2
13	1-Octanol	229	834	1.185	1.167	1.341	0.008	0.006	0.006	1.97E-01	1.01E-07	3.44E-08	-2.3	-23.2	24.8
14	Orcinyl di-angelate	240	798	1.190	1.167	1.341	0.027	0.027	0.027	5.11E-08	3.15E-07	1.07E-07	-24.2	-21.6	-23.1
15	Unknown3	249	919	1.191	1.167	1.341	0.001	0.001	0.001	6.34E-09	3.90E-08	1.33E-08	-27.2	-24.6	-26.2
16	Unknown 4	262	795	1.164	1.167	1.341	0.022	0.019	0.019	1.08E-01	7.01E-08	2.39E-08	-3.2	-23.8	-25.3
17	Decanoic acid	277	763	1.191	1.167	1.341	0.016	0.016	0.016	2.43E-08	1.50E-07	5.11E-08	-25.3	-22.7	-24.2
18	Bicyclo[3.1.1]heptan-3-ol	304	712	1.191	1.167	1.341	0.006	0.006	0.006	1.04E-08	6.40E-08	2.18E-08	-26.5	-23.9	-25.4
19	n-Nonadecanol-1	306	708	1.191	1.166	1.341	0.015	0.015	0.015	9.45E-08	5.82E-07	1.99E-07	-23.3	-20.7	-22.3
20	2-Cyclohexen-1-one	309	859	1.056	1.167	1.341	0.028	0.010	0.010	6.41E-01	5.17E-08	1.77E-08	-0.6	-24.2	-25.8
21	Carvone	312	824	1.191	1.167	1.341	0.014	0.014	0.014	2.61E-09	1.61E-08	5.48E-09	-28.5	-25.9	-27.4
22	Bicyclo[3.1.0]hexan-3-ol, 4-methylene-1-(1-methylethyl)-, acetate	359	764	1.191	1.166	1.341	0.012	0.012	0.012	7.42E-08	4.57E-07	1.56E-07	-23.7	-21.1	-22.6
23	2-Cyclohexen-1-ol	361	952	1.175	1.167	1.341	0.019	0.014	0.014	1.74E-01	2.98E-08	1.02E-08	-2.5	-25.0	-26.6
24	Carveol	370	810	1.156	1.167	1.341	0.033	0.016	0.016	3.31E-01	2.58E-07	8.80E-08	-1.6	-21.9	-23.4
25	Unknown 5	374	825	1.177	1.167	1.281	0.001	0.006	0.005	2.45E-01	1.17E-07	5.74E-01	-2.0	-23.0	-0.8
26	6-Hydroxycarvotanacetone	380	694	1.191	1.166	1.341	0.017	0.017	0.017	3.70E-08	2.28E-07	7.78E-08	-24.7	-22.1	-23.6
27	3-Bromo-5,5-dimethyl-cyclohex-2-enol	387	710	1.341	1.191	1.166	0.019	0.019	0.019	6.01E-07	2.86E-07	1.76E-06	-20.7	-21.7	-19.1
28	1,3,3,4-Tetramethyl-2-oxabicyclo[2.2.0]hexane	400	683	1.191	1.166	1.341	0.018	0.018	0.018	7.55E-08	4.65E-07	1.59E-07	-23.7	-21.0	-22.6
29	Nona-3,5-dien-2-ol	402	700	1.191	1.166	1.341	0.006	0.006	0.006	7.27E-08	4.48E-07	1.53E-07	-23.7	-21.1	-22.6
30	p-Mentha-1 (7),8 (10)-dien-9-ol	404	666	1.158	1.166	1.341	0.040	0.024	0.024	2.39E-01	5.57E-07	1.90E-07	-2.1	-20.8	-22.3
31	Methyl anthranilate	453	889	1.185	1.155	1.341	0.032	0.034	0.031	7.33E-03	4.45E-02	9.34E-10	-7.1	-4.5	-30.0
32	1,2-Cyclohexanediol	461	854	1.169	1.167	1.129	0.000	0.001	0.034	4.80E-01	6.56E-08	8.89E-01	-1.1	-23.9	-0.2
33	Dimethyl phthalate	464	954	1.191	1.166	1.340	0.038	0.038	0.038	1.43E-07	8.83E-07	3.01E-07	-22.7	-20.1	-21.7
34	Unknown 6	474	796	1.191	1.166	1.341	0.027	0.027	0.027	2.21E-08	1.36E-07	4.64E-08	-25.4	-22.8	-24.4
35	2-Hexanamine	29	699	1.191	1.136	1.341	2.E-04	1.E-03	2.E-03	1.72E+07	3.05	4.92E+07	24.0	1.6	25.6
36	2,3-Pentanedione	47	797	1.191	1.136	1.341	4.E-03	1.E-03	9.E-03	2.53E+07	3.05	1.32E+06	24.6	1.6	20.3
37	Phellandrene	96	853	1.171	1.162	1.324	3.E-04	5.E-03	7.E-04	5.73	15.79	6.92	2.5	4.0	2.8
38	unknown7	108	891	1.191	1.167	1.341	5.E-03	2.E-03	3.E-03	6.68E+07	1.19E+08	3.54E+07	26.0	26.8	25.1
39	trans-Ocimene	109	904	1.179	1.154	1.333	4.E-04	2.E-02	5.E-03	10.26	13.59	25.04	3.4	3.8	4.6
40	Terpinene	113	840	1.191	1.167	1.341	5.E-05	5.E-02	3.E-02	1.53E+08	6.38E+07	1.32E+07	27.2	25.9	23.7
41	unknown8	127	872	1.191	1.167	1.341	2.E-03	1.E-02	1.E-03	7.10E+08	9.78E+06	2.68E+08	29.4	23.2	28.0
42	unknown9	129	882	1.191	1.167	1.341	3.E-03	2.E-02	3.E-02	1.45E+08	9.67E+06	1.24E+08	27.1	23.2	26.9
43	5-Hepten-2-one	147	777	1.191	1.167	1.341	2.E-03	1.E-02	2.E-02	3.40E+08	2.17E+07	2.01E+09	28.3	24.4	30.9
44	1-Pentanol	152	852	1.191	1.136	1.341	3.E-04	1.E-03	1.E-03	1.53E+08	3.05	1.11E+08	27.2	1.6	26.7
45	3-Hexen-1-ol	161	888	1.191	1.167	1.341	7.E-04	9.E-03	3.E-05	1.19E+07	2.13E+08	1.94E+07	23.5	27.7	24.2
46	2-Hexen-1-ol	166	841	1.191	1.136	1.341	7.E-03	1.E-03	3.E-03	1.77E+09	3.05E+00	1.41E+08	30.7	1.6	27.1
47	unknown10	234	798	1.191	1.167	1.341	4.E-03	1.E-02	1.E-03	2.45E+06	2.04E+07	7.43E+06	21.2	24.3	22.8
48	Cyclohexanol	247	647	1.191	1.167	1.341	1.E-03	1.E-02	4.E-04	1.58E+06	1.76E+07	6.08E+06	20.6	24.1	22.5
49	Unknown11	297	734	1.341	1.191	1.136	1.E-03	4.E-03	1.E-03	5.42E+08	1.30E+07	3.05	29.0	23.6	1.6
50	Unknown12	331	711	1.341	1.191	1.167	7.E-06	3.E-03	2.E-02	3.17E+07	4.68E+07	2.73E+08	24.9	25.5	28.0

^1^ID: the only data number of the substance in this qualitative analysis.

^2^Similarity: the degree of material matching between the substance and the standard library in qualitative analysis.

^**3**^VIP: the importance of the variable projection of the substance in the OPLS-DA model of the group.

^4^*P*-VALUE: the *P* value of the substance in the t test of the group.

^5^FOLD CHANGE: the multiplier relationship between the two groups of experiments in this group

^6^LOG_FOLDCHANGE: the logarithm of the FOLD CHANGE to the base 2

### Close correlation existed between UPLC-identified and GC-identified metabolites of interest

**Fig. 8:**
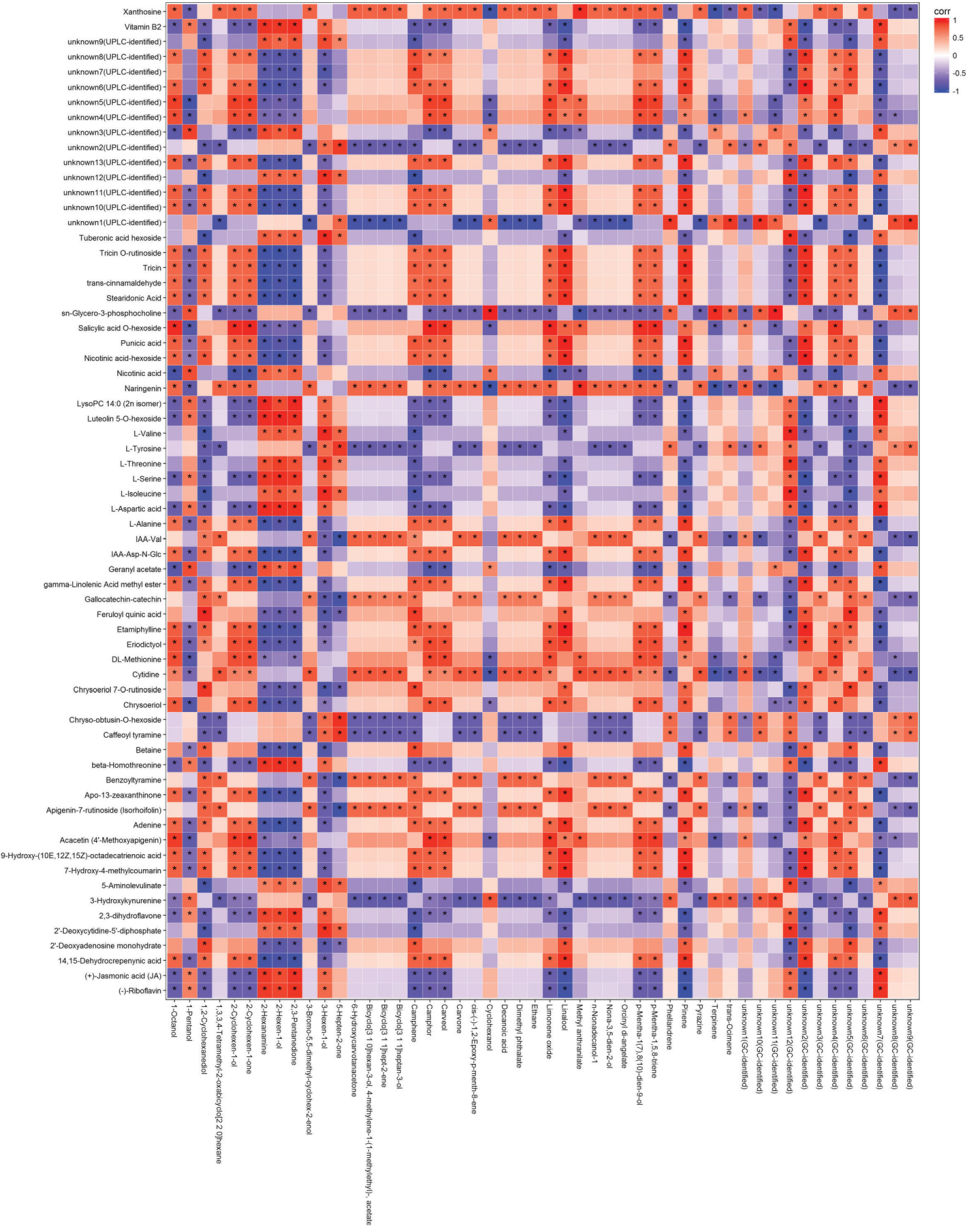
Heatmap illustrating the spearman correlation coefficients between the UPLC-identified and GC-identified metabolites of interest. The white color suggests the correlation coefficient (corr) = 0. The red and blue colors represent positive (0 < corr < 1) and negative (-1 < corr < 0) correlation, respectively. ‘*’ indicates the relationship between the characteristics reached a significant level (*P* < 0.05). Y-axis was the UPLC-identified metabolites of interest generated from UPLC-QQQ-MS and X-axis were the GC-identified metabolites of interest generated from GC-MS

Spearman correlation coefficients between UPLC- and GC-identified metabolites of interest were calculated and the results were illustrated with Heatmap (Fig. 8), which revealed that there were evidently intimate correlations between the two groups of metabolites identified by UPLC-QQQ-MS and GC-MS. The relationship between the characteristics reached a significant level (*P* < 0.05) was indicated by ‘*’ (Fig. 8). Pinene, 2,3-penatedione, 3-hexen-1-ol, 2-hexen-1-ol, 2-hexanamine, linalool, camphene, unknown 2, 5, 7, 12 (GC-identified metabolites), 1,2-cydohexanediol were positively or negatively correlated to over 50 UPLC-identified metabolites of interest (Fig. 8). While xanthosine, sn-glycero-3-phosphocholine, naringenin, cytidine, 3-hydroxykynurenine were correlated to 37 of the selected GC-identified metabolites of interest (Fig. 8).

**Fig. 9:**
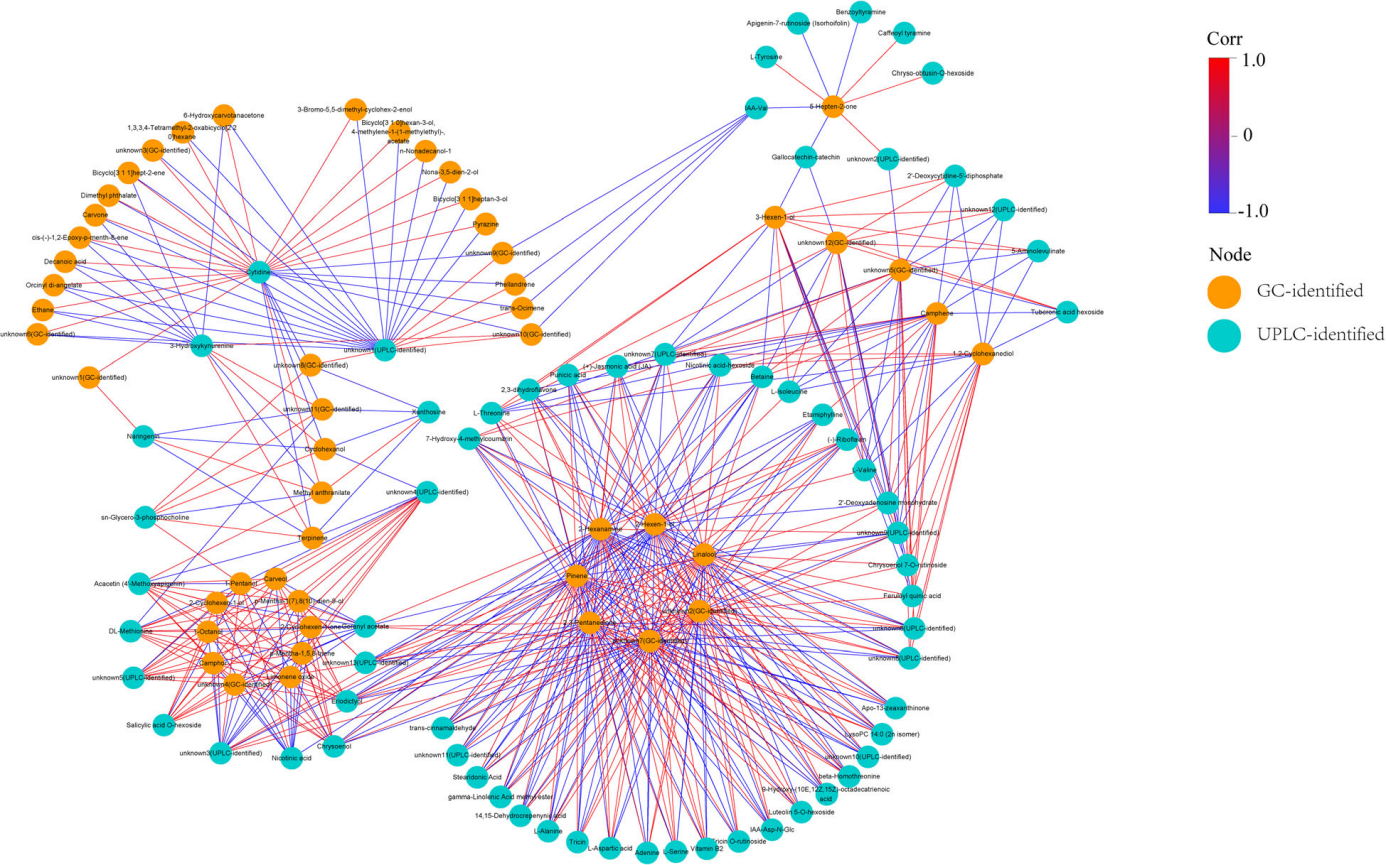
Correlation network of pairs between the UPLC-identified and GC-identified metabolites of interest (corr > 0.8 and *P* < 0.05). Data were processed with Cytoscape (v3.5.1). Orange nodes represent GC-identified metabolites and turquoise nodes represent UPLC-identified metabolites. The red and blue colors of the edges represent positive (0 < corr < 1) and negative (-1 < corr < 0) correlation, respectively

Pairs with the correlation coefficient > 0.8 and the corresponding *P*-value < 0.05 were visualized via Cytoscape (v3.5.1). The correlation network contained 116 nodes and 534 edges (Fig. 9). Generally, UPLC-identified metabolites and GC-identified metabolites were clustered separately. GC-identified pinene, 2-hexen-1-ol, 2-hexanamine, 2,3-penatedione, linalool, unknown 2 and 7 were strongly correlated to 38, 38, 37, 37, 36, 35 and 35 UPLC-identified metabolites of interest, respectively, which formed the biggest cycle in the lower part of Fig. 9. It was noteworthy that those seven GC-identified metabolites with correlation to over 50 UPLC-identified metabolites of interest. And those seven metabolites were divided into two groups: group A (pinene, linalool and unknown 2) and B (2-hexen-1-ol, 2-hexanamine, 2,3-penatedione and unknown 7), which had higher and lower amount in the tender shoots of Chongyi wild mandarin, respectively. The UPLC-identified metabolites that were positively correlated to group A were negatively correlated to group B and vice versa (Additional file 2: Table S1).

As to the UPLC-identified metabolites, cytidine, unknown 1, chrysoeriol, eriodictyol and geranyl acetate were strongly correlated to 27, 22, 16, 16 and 14 GC-identified metabolites of interest, respectively (Fig. 9). Cytidine and unknown 1 (UPLC-identified) had similar correlated metabolites, while chrysoeriol, eriodictyol and geranyl acetate shared similar correlated metabolites.

## Discussion

Now citrus growers rely heavily on the use of pesticide to control ACP, which are costly and cause great environmental concerns. Therefore, visual cues plus repellent or attractant for citrus psyllid control were powerful alternatives in ACP monitoring and control [29]. But the efficiency of this strategy largely depends on the volatile or colored chemicals identified from ACP’ hosts, because they generally rely on vision and olfaction for the selection of hosts [25]. Because flushing shoots can directly influence the oviposition and development of ACP [35, 36], it is easy to infer that more flushing shoots will result in larger amount of ACP to feed on. However, the results of the four-choice tests demonstrated that Hong Kong kumquat had constantly more new shoots, but Chongyi wild mandarin was constantly more attractive to ACP. Therefore, visual and olfactory stimuli generated by tender shoots of Chongyi wild mandarin may be pivotal in the stimulatory response of ACP.

ACP was more attractive to ‘*Ca*. L. asiaticus’-positive plants that were less saturated in yellow color than negative ones, probably due to the fact that infected leaves possessed an altered plane of polarization for yellow (591 nm) wavelengths [27, 37, 38]. These reports were in consistent with our results that the yellow color in the tender leaves of ACP-preferred Chongyi wild mandarin was less saturated. The color of yellowish green leaf is mainly dominated by chlorophyll (Chl) and carotenoid (Car) [39]. The formation of 5-Aminolevulinate (5-ALA), derived from glutamate, is the key step in the biosynthesis of chlorophyll and other four tetrapyrroles [40, 41]. But the content of 5-ALA in Chongyi wild mandarin was significantly lower than that in the other three host plants, which did not result in the lowest total Chl content. However, lower 5-ALA content may lead to lower content of chlorophyllide *a*, an anti-herbivory compound in citrus [42], which may cause Chongyi wild mandarin to attract more ACP. In the case of Car, Chongyi wild mandarin had the lowest Car content and highest apo-13-zeaxanthinone, which was reasonable because apocarotenoids can be produced by carotenoid cleavage dioxygenases (CCDs) [43]. Intriguingly, apocarotenoids are versatile aroma chemicals [44] and the volatile apo-13-zeaxanthinone may be preferred by ACP.

Other two pathways rich in yellow secondary metabolites are flavonoid biosynthesis pathway, flavone and flavonol biosynthesis pathway. Since ACP is attracted to yellow in general [26], it is reasonable that six out of eight identified yellow flavones plus one flavonoid had higher contents in ACP-preferred Chongyi wild mandarin. Besides their yellow color characteristics, flavonoids/flavones/flavonols have been suggested to act as feeding deterrents or stimulants in pest control experiments [45–47]. For instance, unsubstituted flavones/flavonoids like eriodictyol and apigenin were the strongest feeding deterrents in the choice assay of 3rd-instars of bertha armyworm (*Mamestra configurata* Walker), while naringenine, 7,40-dihydroxyflavone and dihydroquercetin functioned as stimulants [46]. However, in the results revealed by UPLC-QQQ-MS, apigenin, eriodictyol and naringenine had higher accumulation in Chongyi wild mandarin, indicating that they may play the role as stimulants to ACP. In addition, 2,3-dihydroxyflavone had lower contents in Chongyi wild mandarin, suggesting it may function as a deterrent. The discrepancy could be explained that a specific flavonoid/flavone/flavonol may have a dual nature as deterrent or stimulant to different pests [45]. The other possibility was that the behavioral response of insects to a compound could vary depending on the concentration and the stage of the insect tested [48].

Along with the color-related metabolites identified by UPLC-QQQ-MS, two phytohormones may also contribute to the preference of ACP to Chongyi wild mandarin: salicylic acid (SA) and jasmonic acid (JA). SA was repeatedly reported to take part in biotic and abiotic stresses [49–51]. JA, the de-methylated product of methyl jasmonate (MeJA), could suppress the conversion of SA into methyl salicylate (MeSA) and MeJA itself could suppress the emission of MeSA [50–52]. The higher content of SA o-hexoside and lower content of JA may all lead to higher amount of MeSA/SA in Chongyi wild mandarin. MeSA turned out to be a key ACP attractant [51, 53], and high level of SA can block the biosynthesis of JA, which is required for wound-inducible systemic defense responses [48, 54]. Moreover, the above-mentioned chlorophyllide *a* was suggested to induce the JA-mediated pathway, which plays a positive role to defend herbivore in citrus [41]. Therefore, the lower content of JA and higher content of MeSA may make Chongyi wild mandarin a more preferred host plant.

In the four-choice test, the host preference of ACP was influenced mainly by visual and olfactory stimuli. Chongyi wild mandarin and ‘Gannan zao’ shared similar color parameters. Apart from visual stimuli, ACP host preference is strongly influenced by volatiles emitting from the host plants [28, 33, 55]. Therefore, olfactory stimuli from Chongyi wild mandarin probably made it more preferable to ACP than ‘Gannan zao’. This hypothesis has been proved in the following olfactometer assay, strongly indicating that some volatile compounds from the tender shoot of Chongyi wild mandarin could attract *D. citri*.

Monoterpene, together with monoterpene esters and sequiterpenes, commonly exist in the leaves of *Citrus* species [56, 57]. The contents of monoterpenes in Chongyi wild mandarin, such as pinene, camphor, linalool, camphene, carveol, carvone and limonene, were significantly higher than the other three plants, suggesting that they may play as stimulants in affecting the foraging behavior of *D.citri*. However, some of the monoterpenes, like *β*-pinene, linalool and camphor, were suggested to have repellent role in insect behavior [58–60]. Few reports have revealed that linalool, an acyclic monoterpene alcohol, could be used as insect-repellent against corn earworm (*Helicoverpa armigera*), spider mite (*Tetranychus urticae*), mosquito (*Aedes aegypti*) and beetles (*T. castaneum* and *Dominica Rhyzopertha*) [60–62]. But linalool was suggested to function as an attractant at least at the beginning of host plant searching for western flower thrips (*Frankliniella occidentalis*) [63]. And a combination of terpenoids such as linalool, myricene, and pinene appeared to synergistically attract the western (*Diabrotica virgifera virgifera*) and northern corn rootworm beetles (*D. barberi*) [64]. In addition, there was a Chinese patent (NO. 104222078A) applied by South China Agricultural University revealed that a combination of γ-terpinene, α-pinene plus linalool could trap significantly more *D. citri* than control, indicating that the proper concentration and combination of terpenoids or VOCs in Chongyi wild mandarin may be attractive to ACP. Moreover, *D. citri* may prefer these monoterpenes because they are prevalent in Rutaceae and may represent the presence of flush, which is pivotal for the mating, oviposition and development of *D. citri* [36, 55].

Correlation analysis revealed that close relationship existed between UPLC-identified and GC-identified metabolites of interest. The positively and negatively correlated metabolites may have synergistic and antagonistic effect on each other, which may work together to affect the host preference of *D. citri*. For example, *β*-ocimene synthase in the terpenoid pathway is under the control of the JA pathway [65], which agreed with our result that trans-ocimene was positively correlated to JA but negatively correlated to SA o-hexoside because negative cross-talk exists between the JA and SA pathways in citrus plants [66]. Moreover, exogenous application of MeJA and SA enhanced and suppressed the amount of E-*β*-ocimene, namely trans-ocimene, respectively, which was suggested to be a volatile attractive to many herbivores’ natural enemies [51, 67]. It is acceptable that host plants with lower amount of volatiles attractive to its natural enemy will be more attractive to *D. citri*, such as Chongyi wild mandarin.

## Conclusions

Taken together, Chongyi wild mandarin was more attractive to ACP than the other three host plants, indicating a great potential of wild citrus germplasm in HLB-related study. Metabolic profiling of the tender shoots from the four Rutaceae plants suggested four possible reasons. The first reason may be the less saturated yellow color of the tender shoot. The second reason may be that Chongyi wild mandarin contained more ACP-preferred metabolites, such as MeSA, apocarotenoids as well as some monoterpenes. The third reason may be lower amount of repellents, such as the compounds favored by ACP’s natural enemies (trans-ocimene) and the metabolites playing anti-herbivory role (chlorophyllide *a*). The fourth reason may be due to the insensitivity of JA-mediated wouding response from the host plant, which will generate negative influence on ACP’s development or oviposition. Even though ACP host plant preference is based on complex profile combinations, our results may, with further investigations, contribute to the elucidation of the underlying mechanism.

## Methods

### Plant materials

Four host plants of Asian citrus psyllid (ACP, *Diaphorina citri*) were analyzed in our experiments: Chongyi wild mandarin (C), orange jasmine (J, *Murraya exotica* L.), ‘Gannan zao’ navel orange (G, *Citrus sinensis*) and wild Hong Kong kumquat (Y, *Fortune hindisii* Swingle). They are all Rutaceae host of “*Candidatus* Liberibacter asiaticus” (*C*Las). Seeds of orange jasmine were purchased from a certified seed company in Jiangsu, China. Seeds of Chongyi wild mandarin and wild Hong Kong kumquat were collected from the mountains of Chongyi county and Anyuan county of Ganzhou, China, respectively. ‘Gannan zao’ is a new early-ripening cultivar released by National Navel Orange Engineering Research Center (NORC), China, which is grafted onto *Poncirus triafoliata* rootstock for propagation and the other three tested plants are seedlings. The potted plants were cultured in the nursery of NORC for one and a half years. The *C*Las-negative plants were moved to the negative pressure laboratory at 25±3°C with 14 h:10 h (light:dark) photoperiod cycle (60±5% relative humidity, RH) and cultured for half a year before use. Two-year-old plants were used for host preference of ACP and the following experiments. Tender shoots of uninfested plants were sampled, immediately frozen in liquid nitrogen and stored at -80 ^o^C till use.

### Asian Citrus Psyllids

*Diaphorina citri* adults were obtained from a laboratory-reared colony at NORC. The colony was established using psyllid eggs laid by adults collected from a mature navel orange block in 2012. Regular PCR (every two month) proved that the offspring is *C*Las-negative. And the culture was maintained on a mixture of caged sweet orange (*C. sinensis* (L.) Osbeck.) and orange jasmine in the negative pressure laboratory mentioned above.

### Four-choice test

Half a month before host preference analysis, heading-back cut was applied to promote more shooting. Each plant of orange jasmine, Chongyi wild mandarin, wild Hong Kong kumquat and ‘Gannan zao’ were placed in the four corners of the same cage made from polyvinyl chloride tubes (2 cm in diameter) with the size of 75*75*90 (length*width*height). The cage was wrapped up with 0.25 mm diameter nylon net to prevent the spread of psyllids. Another two cages of plants were prepared as replications. A vial of psyllids (about 100) without regard to gender or age was collected from the laboratory-reared colony. After being starved for 1 h, the vial of psyllids was placed on the floor at the center of the cage and let them fly to feed freely. The number of psyllids on each plant was counted without disturbing two days after the release. And then, the psyllids were removed from the plants and another 100 psyllids were released again. The releasing, counting and removing process continued a month to analyze the dynamic host preference situation of psyllids on the four kinds of plants. Because the life cycle of ACP is closely related to the growth pattern of its host plants [36], the numbers of the tender shoots of each tested plants were counted during the same period of four-choice test.

However, host selection is not a static process as the needs of the insect at various developing stages may vary and the suitability of the plant to the insect may vary with time [48]. Therefore, in the one-month-long four-choice test, the plants were unchanged and ACPs were randomly collected regardless of gender and age to consider them as a population.

### *D. citri* olfactometer assay

The preference of *D. citri* toward the four plant species was tested with a 6-arm olfactometer (BL6-300M, Shanghai BILON instrument corporation, China). There were two opposite entries blocked manually because only four samples were investigated and there was no 4-arm olfactometer available in NORC. Because it was the most preferred host of *D. citri* in the four-choice test, the location of the tender shoot from Chongyi wild mandarin was unchanged, while the other three tender shoots were placed next to it one by one to eliminate its peer effect. Individual constant charcoal-filtered air flows at the rate of 0.4 L min^-1^ was connected to each tender shoot. And the air flows contained the odour from each plant were converged to the activity room (35 cm*35 cm*3 cm) with a polytetrafluoroethylene tubes. The tubes were cleaned with the charcoal-filtered air flows when the places of the tender shoots were changed. Two olfactormeter bioassays were conducted. In the first experiment, 80 adult *D. citri* regardless of gender and age, being starved for 1 h before use, were released in the center of the activity room through a lid (6 cm in diameter), whose edge was covered with Vaseline. Ten minutes later, the numbers of insects in different arms were counted. Three replications were done for the first olfactormeter bioassay. In the other experiment, a single adult female, being starved for 1 h before use, was released in the center of the activity room. Ten minutes later, the place of the female *D. citri* was recorded. If the insect stayed in the arm connected to Chongyi wild mandarin, then it scored 1, and so on. Ten replications were performed each day and the dataset from 8 independent days were used in the data analysis. All the experiments were carried out at room temperature, 60±5% RH and constant light (2300 lux).

### Measurement of leaf color parameters, chlorophyll and carotenoid concentration

Color parameters of tender leaves from the four tested plants were measured with a NR200 Precision Colorimeter (Shenzhen 3nh Technology Co., Ltd., China) and quantified using the CIELAB color space system. The values of *L*^∗^, *a*^∗^, and *b*^∗^ explain a 3-dimensional color space. The value of *L*^∗^ is the vertical axis and defines lightness. The values of *a*^∗^ and *b*^∗^ are perpendicular horizontal axes and define red-to-green and blue-to-yellow, respectively. Positive value of *a*^∗^ indicates red color, whereas negative one indicates green. Positive value of *b*^∗^ indicates yellow color and negative one indicates blue. The values of *L*^∗^, *a*^∗^ and *b*^∗^ were obtained directly from the Colorimeter. Chroma (*C*^∗^, color saturation) and Hue (*H*^∗^, color shading) were calculated as follows: *C*^∗^ = (*a*^∗2^ + *b*^∗2^)^1*/*2^ and *H*^∗^ = 180 + tan^-1^ (*b*^∗^/*a*^∗^), (if *a*^∗^ < 0 and *b*^∗^ > 0). The greater the value of *C*^∗^, more saturated the color will be. *H*^∗^ defines colors expressed on 360° grid: 0°= red; 60°= yellow; 120°= green; 180°= cyan; 240°= blue; 300°= magenta [68, 69].

Chlorophyll and carotenoid concentrations were measured as described by Liu et al. (2008) with some modifications [70]. Fresh tender leaves from four uninfested plant materials were randomly collected, respectively. About 0.2 g fine powder of leaf tissue was homogenized in 2 mL of 80% acetone and kept for 15 min at room temperature in dark. The crude extraction was centrifuged at 10, 000 r min^-1^ under room temperature for 20 min, and the supernatant was used for assessing absorbance at 663, 645 and 480 nm with a spectrophotometer (Shimadzu UV-2600, Japan). The concentration (mg/L) of Chlorophyll *a* (*C*_a_), Chlorophyll *b* (*C*_b_), total chlorophyll (*C*_t_) and carotenoid (*C*_x.c_) were calculated as follows: *C*_a_ = 12.21A_665_ – 2.81A_649_, *C*_b_ = 20.13A_649_ – 5.03A_665_, *C*_t_ = *C*_a_ + *C*_b_, *C*_x.c_ = (1000A_470_ – 3.27*C*_a_ – 104*C*_b_)/229.

### UPLC-QQQ-MS analysis

The freeze-dried tender shoots of four plant varieties were crushed using a mixer mill (MM 400, Retsch) with a zirconia bead for 1.5 min at 30 Hz, respectively. Then 100 mg powder was extracted overnight at 4 °C with 1.0 mL 70% methanol containing 0.1 mg L^-1^ lidocaine as internal standard. The mixture was vortexed for three times at intervals. After being centrifuged at 10000 g for 10 min, the supernatant was filtrated with organic phase needle filter 0.22-μm pore size (SCAA-104, ANPEL, Shanghai, China) before LC/MS analysis.

The extracts were analyzed using an LC-ESI-MS/MS system (HPLC, Shim-pack UFLC SHIMADZU CBM20A system, Japan; MS, Applied Biosystems 4000 QTRAP, USA). The separation was achieved using a VP-ODS C18 column (particle size 5.0 μm, 2 mm × 150 mm; Shim-pack, Japan). The mobile phase consisted of 0.04% acetic acid in water and 0.04% acetic acid in acetonitrile. The gradient elution program was set as follows: water: acetonitrile, 95:5 V/V at 0 min, 5:95 V/V at 11.0 min, 5:95 V/V at 12.0 min, 95:5 V/V at 12.1 min, 95:5 V/V at 15.0 min. The flow rate, temperature and injection volume were 0.4 mL min^-1^, 40 °C, 5 μL, respectively.

Linear ion trap and triple quadrupole scans were acquired on a 4500 Q TRAP® LC/MS/MS System (API, USA) equipped with an ESI-Turbo Ion-Spray interface. The ESI source operation parameters were set as follows: source temperature, 550 °C; ion spray voltage, 5500 V; curtain gas, 25.0 psi; the collision gas, high.

### GC-MS analysis

Solid-phase microextraction (SPME) combined with gas chromatography-mass spectrometry (GC-MS)-based volatiles profiling was well-known for its high efficiency in the identification of volatile aroma compounds [71]. The freeze-dried tender shoot was taken into the 20 mL headspace vial. In the solid phase microextraction cycle of CTC rail system (CTC Analytics, Switzerland), the extraction temperature was kept at 50 °C and the vials were shaken for 15 min at the rate of 250 r/m. The fiber assembly was 50/30 μm DVB/CAR on PDMS and the extraction time was 30 min. After desorption for 4 min, the volatiles were used for GC-MS analysis directly, which was performed on an Agilent 7890 gas chromatograph system coupled with a Agilent 5975C mass spectrometer. The system utilized a DB-Wax capillary column with 30 m×250 μm inner diameter and 0.25 μm film thickness (J&W Scientific, Folsom, CA, USA). The analyte was injected in a splitless mode. Helium was used as the carrier gas with the front inlet purge flow 3 mL min^-1^. And the gas flow rate through the column was 1 mL min^-1^. The initial temperature was kept at 40 ^o^C for 5 min, then raised to 250 ^o^C at a rate of 5 °C min^-1^, and then kept at 250 ^o^C for 5 min. The injection, transfer line, ion source and quad temperatures were 260 ^o^C, 260 ^o^C, 230 ^o^C, and 150 ^o^C respectively. The energy was – 70 eV in electron impact mode. The mass spectrometry data were acquired in full-scan mode with the m/z range of 33-459 after a solvent delay of 0 s.

### Data analysis

The data were analyzed via SAS software package (version 9.4, SAS Institution, USA). Statistical difference was compared based on Duncan’s multiple range-tests at the significance level of *P* < 0.05 or 0.01.

UPLC-QQQ-MS data analysis was identified through matching accurate mass and tandem MS/MS spectra with in-house metabolite tandem MS/MS databases MWDB (metware database). Features of isotopes, adducts and mass fragments were removed in the identification of some metabolites. Metabolites annotation was applied with MassBank (http://www.massbank.jp/), KNAPSAcK (http://kanaya.naist.jp/KNApSAcK/), HMDB (http://www.hmdb.ca/), MoTo DB (http://www.ab.wur.nl/moto/), and METLIN (http://metlin.scripps.edu/index.php). The quantification of metabolites were based on multiple reaction monitoring method [72] and Analyst 1.6.1® software (SCIEX, USA). For the identification of metabolites from GC-MS analysis, Chroma TOF 4.3X software of LECO Corporation and NIST database were used for raw peaks exacting, the data baselines filtering and calibration of the baseline, peak alignment, deconvolution analysis, peak identification and integration of the peak area.

All the resulted three-dimensional data involving the peak number, sample name, and normalized peak area were fed to SIMCA14.1 software package (MKS Data Analytics Solutions, Umea, Sweden) for principal component analysis (PCA) and orthogonal projections to latent structures-discriminate analysis (OPLS-DA). To refine the analysis, the first principal component of variable importance in the projection (VIP) was obtained. The VIP values exceeding 1.0 were first selected as changed metabolites. In addition, the remaining variables were assessed by Student's *t*-test (*P*-value > 0.05) and variables were discarded between two comparison groups. Namely, the condition of selecting the differential metabolites was VIP > 1 and *P*-value < 0.05. Moreover, commercial databases including KEGG (http://www.genome.jp/kegg/) and MetaboAnalyst (http://www.metaboanalyst.ca/) as utilized to search for the pathways of metabolites.

Spearman correlation coefficient was used to evaluate the correlation between the GC-identified and UPLC-identified metabolites of interest. Those metabolite pairs with the correlation coefficient > 0.8 and the corresponding *P*-value < 0.05 were processed via Cytoscape (v3.5.1) to make the correlation network.

## Additional files

Additional file 1: **Figure S1-S4**. (DOCX 990 kb)

Additional file 2: **Table S1.** The correlation coefficient of seven GC-identified metabolites with some UPLC-identified metabolites.. (DOCX 18 kb)

## Abbreviations

−*a*^*^: greenness
ACP: Asian citrus psyllid
+*b*^*^: yellowness
C: Chongyi wild mandarin
*C*^*^: Color saturation
*C*_a_: Content of chlorophyll *a*
Car: Carotenoid
Chl: Chlorophyll
*C*_b_: Content of chlorophyll *b*
*C*_t_: Content of total chlorophyll
*C*_x.c_: Content of carotenoid
*C*Las: *Candidatus* Liberibacter asiaticus
Corr: Correlation coefficient
*D.* citri: *Diaphorina citri*
5-ALA: 5-aminolevulinate
G: ‘Gannan zao’ navel orange
*H*^∗^: color shading
HLB: huanglongbing
GC-MS: gas chromatography-mass spectrometry
J: Orange jasmine
JA: Jasmonic acid
KEGG: Kyoto encyclopedia of genes and genomes
*L*^*^: Lightness of the color
MeJA: Methyl jasmonate
MeSA: Methyl salicylate
NORC: National Navel Orange Engineering Research Center
OPLS-DA: Orthogonal projections to latent structures-discriminate analysis
PCA: Principal component analysis
PCR: Polymerase chain reaction
RH: Relative humidity
SA: Salicylic acid
SAS: Statistical analysis system
SPME: Solid-phase microextraction
UPLC-QQQ-MS: Ultra high performance liquid chromatography coupled to triple quadrupole tandem mass spectrometry
VIP: First principal component of variable importance in the projection
VOCs: Volatile compounds
Y: Wild Hong Kong kumquat
