# Supplementary material for: Effects of leaf colorness, pigment contents and allelochemicals on the orientation of the Asian citrus psyllid among four Rutaceae host plants
Source: BMC Plant Biol. 2019 Jun 13;19:254. doi: 10.1186/s12870-019-1818-7 (PMC6567656; doi:10.1186/s12870-019-1818-7)
Supplement: Supplementary file 1 — Figure S1-S4. (DOCX 990 kb) [file 12870_2019_1818_MOESM1_ESM.docx]

**Additional file 1**

**Figure S1**
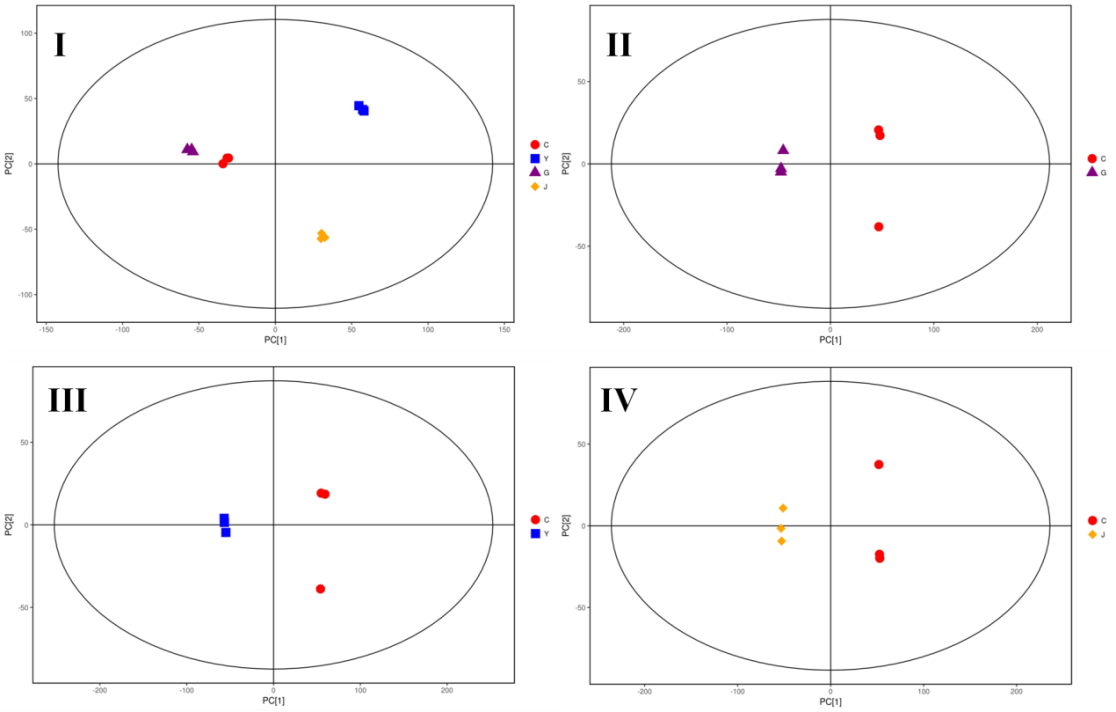


**Figure S1** Score scatter plots for principal component analysis model of UPLC-QQQ-MS analysis. I: total of Chongyi wild mandarin (C), ‘Gannan zao’ navel orange (G), orange jasmine (J), wild Hong Kong kumquat (Y); II: G/C; III: Y/C; IV: J/C.

**Figure S2**


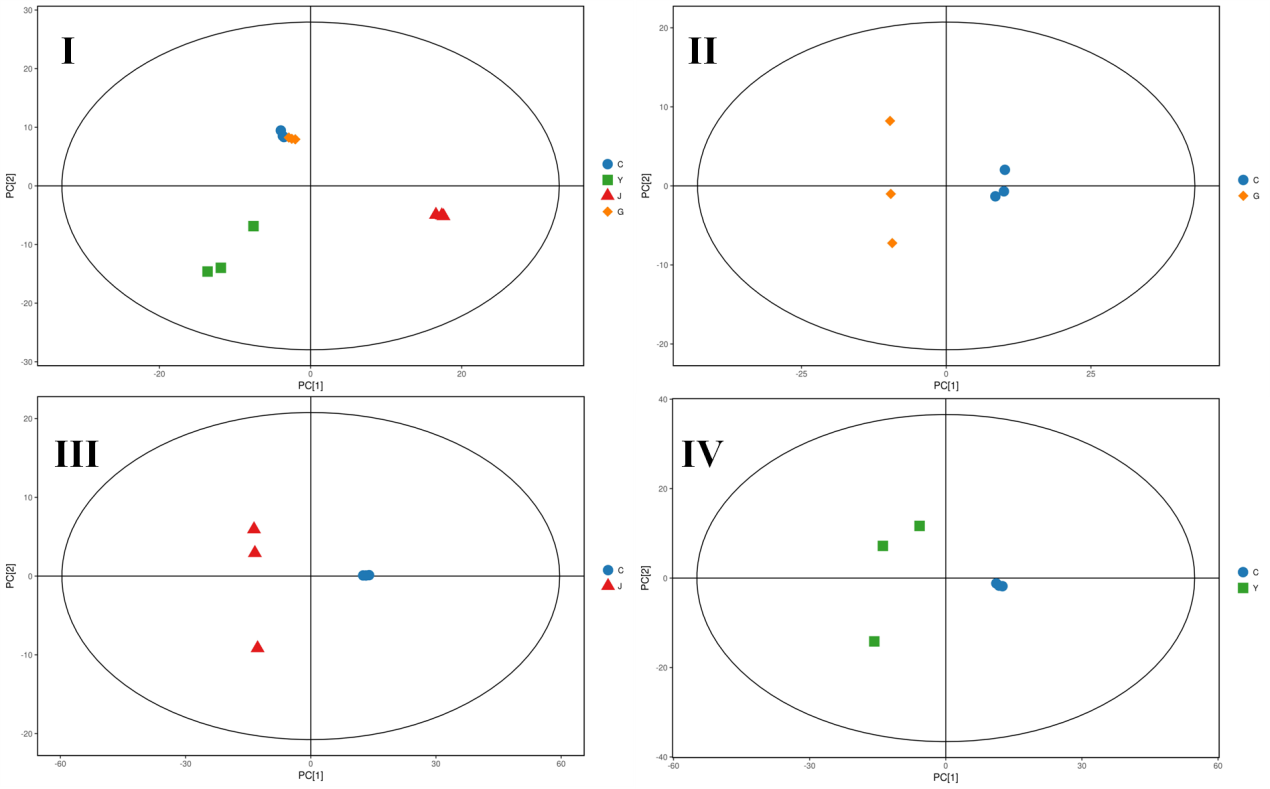


**Figure S2** Score scatter plots for principal component analysis model of GC-MS analysis. I: total of Chongyi wild mandarin (C), ‘Gannan zao’ navel orange (G), orange jasmine (J), wild Hong Kong kumquat (Y); II: G/C; III: Y/C; IV: J/C.

**
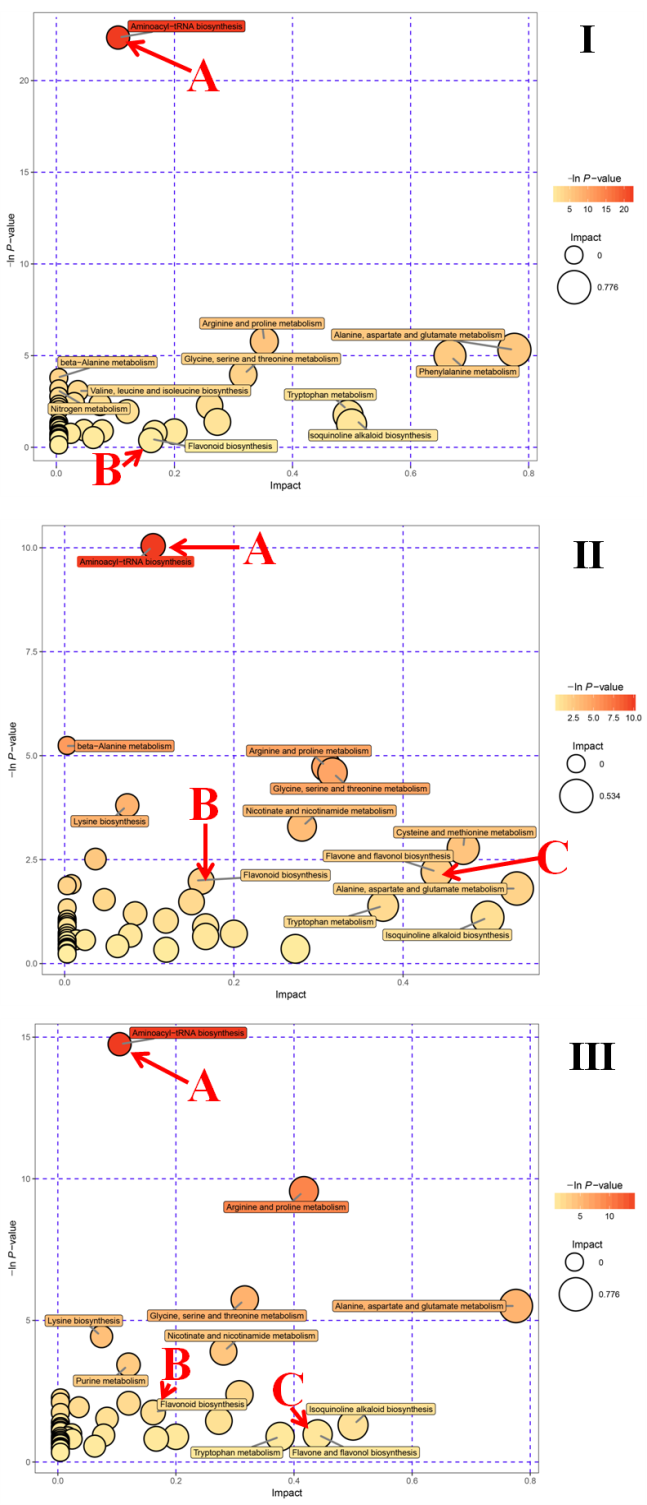
Figure S3**

**Figure S3** Bubble plots of UPLC-QQQ-MS analysis for ‘Gannan zao’ navel orange (I), wild Hong Kong kumquat (II), orange jasmine (III) when compared with Chongyi wild mandarin. Each bubble represents a metabolic pathway. The impact value determines the size of the bubble and the smaller the *P*-value, the deeper the color. A: Aminoacyl-tRNA biosynthesis pathway; B: Flavonoid biosynthesis pathway; C: Flavone and flavonol biosynthesis pathway.

**Figure S4**

**
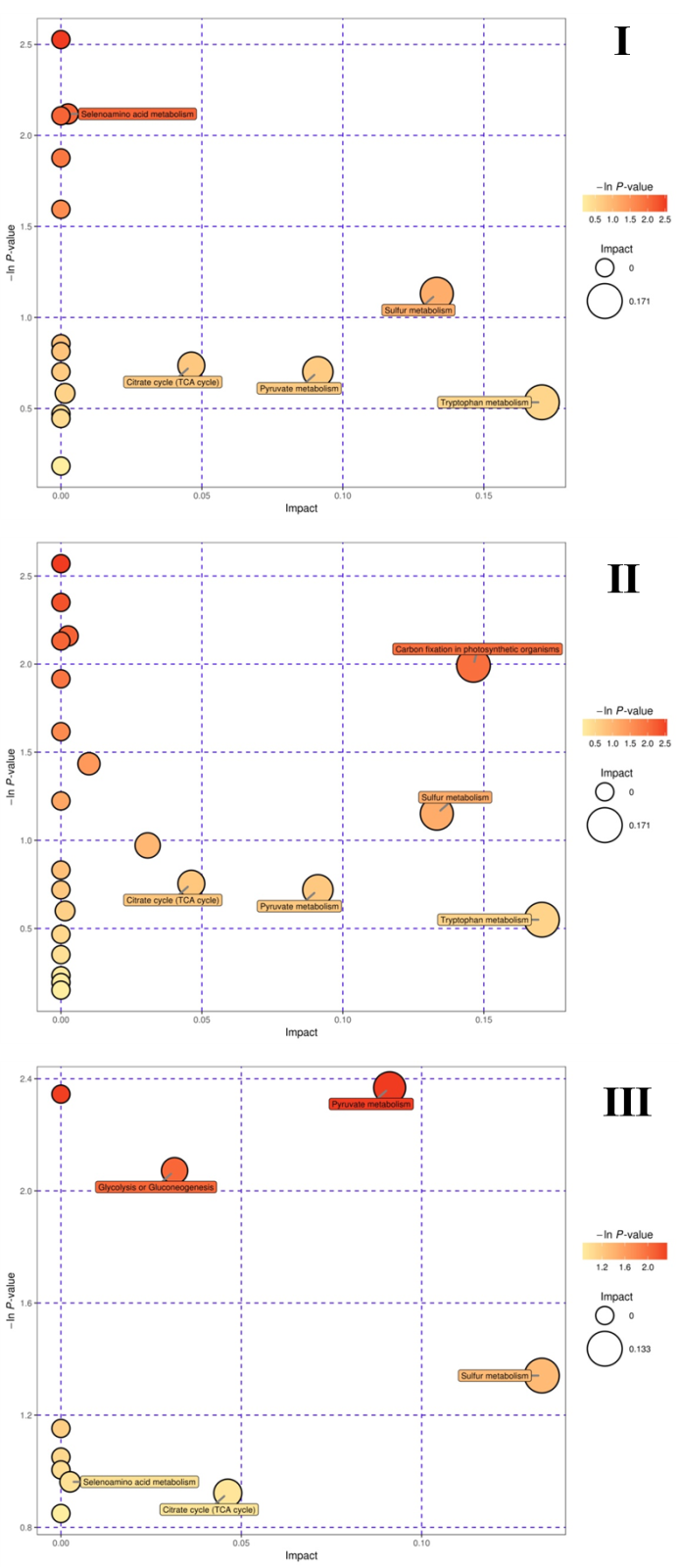
**

**Figure S4** Bubble plots of GC-MS analysis for ‘Gannan zao’ navel orange (I), wild Hong Kong kumquat (II), orange jasmine (III) when compared with Chongyi wild mandarin. Each bubble represents a metabolic pathway. The impact value determines the size of the bubble and the smaller the *P*-value, the deeper the color.
