# Supplementary material for: Effects of leaf colorness, pigment contents and allelochemicals on the orientation of the Asian citrus psyllid among four Rutaceae host plants
Source: BMC Plant Biol. 2019 Jun 13;19:254. doi: 10.1186/s12870-019-1818-7 (PMC6567656; doi:10.1186/s12870-019-1818-7)
Supplement: Supplementary file 2 — Table S1. The correlation coefficient of seven GC-identified metabolites with some UPLC-identified metabolites.. (DOCX 18 kb) [file 12870_2019_1818_MOESM2_ESM.docx]

**Additional file 2 : Table S1.** The correlation coefficient of seven GC-identified metabolites with some UPLC-identified metabolites.

|  | **2-Hexen-1-ol** | **2-Hexanamine** | **2,3-Pentanedione** | **unknown7(GC-identified)** | **Pinene** | **Linalool** | **unknown2(GC-identified)** |
| --- | --- | --- | --- | --- | --- | --- | --- |
| Adenine | -0.96 | -0.93 | -0.94 | -0.93 | 0.94 | 0.94 | 0.95 |
| Tricin O-rutinoside | -0.96 | -0.95 | -0.94 | -0.93 | 0.92 | 0.94 | 0.95 |
| IAA-Asp-N-Glc | -0.94 | -0.96 | -0.96 | -0.94 | 0.91 | 0.92 | 0.93 |
| Nicotinic acid-hexoside | -0.93 | -0.90 | -0.87 | -0.91 | 0.92 | 0.97 | 0.98 |
| 14,15-Dehydrocrepenynic acid | -0.92 | -0.95 | -0.96 | -0.93 | 0.94 | 0.94 | 0.93 |
| Stearidonic Acid | -0.92 | -0.95 | -0.96 | -0.93 | 0.94 | 0.94 | 0.93 |
| Tricin | -0.92 | -0.95 | -0.94 | -0.97 | 0.96 | 0.94 | 0.95 |
| 7-Hydroxy-4-methylcoumarin | -0.91 | -0.94 | -0.92 | -0.96 | 0.97 | 0.96 | 0.97 |
| 9-Hydroxy-(10E,12Z,15Z)-octadecatrienoic acid | -0.91 | -0.90 | -0.92 | -0.90 | 0.97 | 0.97 | 0.97 |
| trans-cinnamaldehyde | -0.91 | -0.96 | -0.96 | -0.94 | 0.91 | 0.94 | 0.93 |
| unknown6(UPLC-identified) | -0.90 | -0.90 | -0.87 | -0.92 | 0.92 | 0.97 | 0.98 |
| Apo-13-zeaxanthinone | -0.90 | -0.94 | -0.94 | -0.96 | 0.93 | 0.94 | 0.95 |
| unknown10(UPLC-identified) | -0.90 | -0.94 | -0.95 | -0.96 | 0.93 | 0.94 | 0.94 |
| unknown11(UPLC-identified) | -0.90 | -0.92 | -0.91 | -0.94 | 0.94 | 0.97 | 0.98 |
| Etamiphylline | -0.90 | -0.91 | -0.92 | -0.93 | 0.98 | 0.99 | 0.97 |
| gamma-Linolenic Acid methyl ester | -0.88 | -0.94 | -0.94 | -0.96 | 0.95 | 0.96 | 0.94 |
| unknown13(UPLC-identified) | -0.87 | -0.90 | -0.92 | -0.87 | 0.96 | 0.94 | 0.92 |
| unknown7(UPLC-identified) | -0.87 | -0.85 | -0.88 | \ | 0.86 | 0.81 | \ |
| unknown8(UPLC-identified) | -0.87 | -0.84 | -0.87 | -0.87 | 0.93 | 0.94 | 0.94 |
| Chrysoeriol | -0.87 | -0.89 | -0.87 | -0.87 | 0.83 | 0.80 | 0.80 |
| L-Alanine | -0.86 | -0.93 | -0.94 | -0.90 | \ | 0.88 | 0.87 |
| Punicic acid | -0.86 | -0.92 | -0.92 | -0.94 | 0.97 | 0.98 | 0.97 |
| Betaine | -0.85 | -0.88 | -0.93 | -0.87 | 0.87 | 0.81 | \ |
| 2'-Deoxyadenosine  monohydrate | -0.85 | \ | \ | \ | \ | 0.85 | 0.85 |
| Eriodictyol | -0.85 | -0.86 | -0.85 | -0.86 | 0.84 | 0.92 | 0.91 |
| unknown3(UPLC-identified) | 0.80 | 0.87 | 0.87 | 0.85 | -0.83 | \ | \ |
| L-Valine | 0.81 | \ | \ | \ | \ | \ | \ |
| L-Isoleucine | 0.83 | \ | \ | \ | \ | \ | \ |
| LysoPC 14:0  (2n isomer) | 0.87 | 0.97 | 0.97 | 0.97 | -0.90 | -0.89 | -0.87 |
| (+)-Jasmonic acid (JA) | 0.87 | 0.93 | 0.95 | 0.95 | -0.96 | -0.95 | -0.94 |
| 2,3-dihydroflavone | 0.88 | 0.89 | 0.93 | 0.90 | -0.94 | -0.89 | -0.87 |
| (-)-Riboflavin | 0.90 | 0.92 | 0.94 | 0.94 | -0.99 | -0.96 | -0.95 |
| L-Threonine | 0.91 | 0.83 | 0.85 | \ | -0.80 | \ | \ |
| Vitamin B2 | 0.93 | 0.94 | 0.96 | 0.92 | -0.97 | -0.94 | -0.93 |
| L-Aspartic acid | 0.95 | 0.97 | 0.94 | 0.94 | -0.92 | -0.93 | -0.94 |
| beta-Homothreonine | 0.95 | 0.94 | 0.93 | 0.92 | -0.95 | -0.94 | -0.96 |
| L-Serine | 0.95 | 0.93 | 0.92 | 0.95 | -0.96 | -0.95 | -0.97 |
| Luteolin 5-O-hexoside | 0.97 | 0.96 | 0.95 | 0.94 | -0.93 | -0.92 | -0.94 |
| unknown9(UPLC-identified) | \ | 0.83 | 0.83 | 0.83 | -0.80 | -0.83 | -0.80 |
| Geranyl acetate | \ | 0.85 | 0.85 | 0.83 | -0.85 | \ | \ |
| Nicotinic acid | \ | \ | \ | \ | -0.82 | \ | \ |
| Feruloyl quinic acid | \ | \ | \ | \ | \ | 0.83 | 0.83 |
| Chrysoeriol 7-O-rutinoside | \ | \ | \ | \ | \ | \ | 0.81 |
